# Boosting the peripheral immune response in the skeletal muscles improved motor function in ALS transgenic mice

**DOI:** 10.1016/j.ymthe.2022.04.018

**Published:** 2022-04-27

**Authors:** Maria Chiara Trolese, Carlotta Scarpa, Valentina Melfi, Paola Fabbrizio, Francesca Sironi, Martina Rossi, Caterina Bendotti, Giovanni Nardo

**Affiliations:** 1 Laboratory of Molecular Neurobiology, Department of Neuroscience, Istituto di Ricerche Farmacologiche Mario Negri Mario Negri IRCCS, Via Mario Negri 2, 20156 Milan, Italy

**Keywords:** amyotrophic lateral sclerosis, mouse models, skeletal muscle, immune cells, satellite cells, myogenesis, motor neuron

## Abstract

Monocyte chemoattractant protein-1 (MCP1) is one of the most powerful pro-inflammatory chemokines. However, its signaling is pivotal in driving injured axon and muscle regeneration. We previously reported that MCP1 is more strongly upregulated in the nervous system of slow-progressing than fast-progressing SOD1^G93A^ mice, the latter showing a poor immune response and eventual massive nerve and muscle degeneration. To assess the MCP1-mediated therapeutic role, we boosted the chemokine along the motor unit of the two SOD1^G93A^ models through a single intramuscular injection of a scAAV9 vector engineered with the *Mcp1* gene. We provided direct evidence underlying the pivotal role of the immune response in driving skeletal muscle regeneration and thus the speed of ALS progression. The comparative study performed in fast- and slow-progressing SOD1^G93A^ mice spotlights the nature and temporal activation of the inflammatory response as limiting factors to preserve the periphery and interfere with the disease course. In addition, we recorded a novel pleiotropic role of MCP1 in promoting peripheral axon regeneration and modulating neuroinflammation, ultimately preventing neurodegeneration. Altogether, these observations highlight the immune response as a key determinant for disease variability and proffer a reasonable explanation for the failure of systemic immunomodulatory treatments, suggesting new potential strategies to hamper ALS progression.

## Teaser

Nardo et al. assessed the MCP1 boosting therapeutic role in a fast- and slow-progressing SOD1^G93A^ mice motor unit. The comparative analysis spotlights the immune muscle response nature and temporal activation as limiting factors to prevent dying-back degeneration. In addition, an MCP1 immune-unrelated role was observed in the nervous system, ultimately preventing neurodegeneration.

## Introduction

Amyotrophic lateral sclerosis (ALS) is a fatal motor neuron (MN) disease characterized by degenerative changes in upper and lower motor neurons. All over the world, it is one of the most common neuromuscular disorders across all ethnicities, with an incidence of 2–3 cases per 100,000 individuals per year.^1^ Onset typically occurs in late middle life and presents as relentlessly progressive muscle atrophy and weakness, with the effects on respiratory muscles limiting survival to 2–4 years after disease onset in most cases.^1^ The diagnosis is based on the clinical assessment of symptoms, with a delay of more than 1 year from symptom onset, quite beyond the therapeutic window of disease-modifying drugs.^1^ Approximately 10% of patients have a familial form of the disease, and mutations in 20 different genes have been associated with the development of ALS. The genes most frequently implicated are *c9orf72, sod1*, *fus*, and *tardbp*. The remaining 90% of cases are sporadic, with an unknown etiology.^2^

The main obstacle to gain full insight into the pathogenesis of ALS is the remarkable clinical heterogeneity of the disease phenotype and course, even in patients carrying the same mutation,^3^^,^^4^ and the multisystemic nature of ALS pathology, which distinctively encompasses distant biological systems, making the identification of a proper therapeutic target even more challenging.^5^^,^^6^

Over the last 20 years, the use of mutant SOD1 (mSOD1) mice has allowed the identification of several pathogenic mechanisms downstream SOD1 gene mutations, which contribute to MN injury within the CNS.^7^ However, this remarkable body of knowledge did not yield the expected outcomes in terms of therapeutic benefits to ALS patients. This evidence suggests that MN protection alone is insufficient to prevent peripheral axons and muscles from degenerating. Indeed, both pharmacological and genetic interference with apoptotic pathways in the MN cell body only marginally affects the lifespan of mSOD1 mice.^8^^,^^9^

Muscle weakness is one of the hallmarks of ALS.^1^^,^^2^ In transgenic mSOD1 mice, which recapitulate the lower MN degeneration, muscle denervation atrophy occurs before any clear signs of neurodegeneration.^10^^,^^11^ This evidence has led to ALS being hypothesized as a distal axonopathy whereby skeletal muscles actively contribute to a retrograde signaling cascade that culminates with the MN death.^12^ Moreover, it has emerged that certain aspects of ALS are non-cell autonomous and that other cell types within the spinal cord, including microglia, astrocytes, and T cells, contribute to the progression of the disease.^13^^,^^14^

Mounting evidence has highlighted the distinct contribution of the inflammatory response in the central nervous system (CNS) with respect to the periphery (i.e., nerves and muscles) in ALS.^15^^,^^16^ Indeed, while the aberrant glial cells’ activation, T cell infiltration, and the resulting release of pro-inflammatory molecules drive neurodegeneration, successful axon and muscle regeneration depends on the coordinated efforts of immune cells that, besides removing cellular debris, release factors that support wound healing.^17^, ^18^, ^19^, ^20^ This may explain the association between the peripheral nervous system (PNS) inflammation and the longer disease duration recently observed in ALS patients with SOD1 mutation.^21^

This evidence indicates that the immune response can actively influence the disease progression, promoting the phenomena of protection and/or toxicity.^22^^,^^23^ Therefore, shed light on the temporal and mechanistic involvement of the immune response in the different compartments affected by the disease will be a practical approach to discover new biomarkers and identify targets for developing precise therapeutic strategies to ameliorate ALS progression.

We recently characterized two mouse strains (C57 and 129Sv) carrying the same copies of human mutant SOD1 transgene (SOD1^G93A^) but exhibiting remarkable differences in terms of disease progression and overall survival.^24^^,^^25^ We found that, despite the same extent of MN loss during disease progression,^24^ fast-progressing ALS mice (129SvSOD1^G93A^) showed earlier muscle denervation and higher axonal dysregulation that correlated with a poor inflammatory response and reduced macrophage infiltration in the periphery^26^^,^^27^ compared with the slow-progressing ALS mice (C57SOD1^G93A^). Further analyses showed that fast-progressing mSOD1 mice failed to activate monocyte chemoattractant protein 1 (MCP1) in MN perikarya and peripheral axons compared to C57SOD1^G93A^ mice.^26^^,^^28^ This evidence suggests that MCP1 signaling and immune cell recruitment may be pivotal in delaying muscular denervation and triggering regeneration in the PNS,^29^^,^^30^ thus regulating the speed of the disease progression of the two ALS models.

MCP1 is an 8-kDa secretory protein, usually released to exert a potent pro-inflammatory effect by binding the specific CCR2 on its target cells. MCP1/CCR2-mediated signaling drives the downstream phosphatidylinositol 3-kinase/Akt and mitogen-activated protein kinase (MAPK) pathways, and it is known that this axis induces chemotaxis of monocytes/macrophages,^31^^,^^32^ microglia,^33^ lymphocytes,^34^, ^35^, ^36^ and neutrophils,^37^ leading to pathological microgliosis and inflammatory activation in chronic disorders.^38^

MCP1 levels are increased in serum and the cerebrospinal fluid of sporadic and familial ALS patients.^39^ In addition, studies in mSOD1 mice have shown that MCP1 is significantly upregulated in the spinal cord and peripheral nerves at the early disease stage, suggesting a pathogenic role for this chemokine.^26^^,^^40^ Nonetheless, evidence has depicted MCP1 as a neuroprotective factor involved in modulating blood-brain barrier permeability,^41^ promoting the differentiation of neural progenitors^42^^,^^43^ and axonal elongation.^44^^,^^45^ Moreover, the chemotactic activity of MCP1 toward macrophages and T cells is crucial in wound healing and the regenerative processes of nerves^46^, ^47^, ^48^ and muscles^29^^,^^49^, ^50^, ^51^, ^52^, ^53^ following acute trauma.

While the effective role of immune cell recruitment in the PNS is still controversial,^15^^,^^16^^,^^54^^,^^55^ no experimental inference is available on the immune response in skeletal muscles during ALS course.

In the present study, we investigated the therapeutic efficacy of a scAAV9 vector engineered with the *Mcp1* gene injected in the skeletal muscles of fast- and slow-progressing SOD1^G93A^ mice. We found that an early boosting of immune response in the peripheral compartment is crucial in countering the denervation atrophy and slowing down the ALS progression in slow- but not fast-progressing mSOD1 mice. Moreover, our data described a pleiotropic role of MCP1 in the CNS as a protective factor able to modulate the neuroinflammation, possibly reducing MN loss. This evidence is instrumental in comprehending the contribution of the immune response in ALS, shedding light on its worth in governing the speed of the disease progression.

## Results

### MCP1 is more expressed in the CNS and PNS of C57SOD1^G93A^ than 129SvSOD1^G93A^ mice

We previously found that MCP1 was significantly upregulated by MNs and peripheral axons of slow-progressing than fast-progressing SOD1^G93A^ mice at disease onset.^26^^,^^28^ The histological analysis supported the higher activation of MCP1 in the spinal cord of C57SOD1^G93A^ compared with the 129SvSOD1^G93A^ model (Figures S1A and S1B), confirming the chemokine expression by MNs (Figures S1C, S1D, and S2A–S2C) and microglia (Figures S2D–S2F) but not astrocytes (Figures S2G–S2I) in the CNS of mSOD1 mice.^40^ Notably, a temporal change in the chemokine expression pattern was found in C57SOD1^G93A^ as the disease progresses, characterized by a significant MCP1 expression by microglia and MNs at the pre-symptomatic and onset disease stage, respectively, followed by a widespread activation at the advanced phases (Figure S1E). Immunofluorescence analysis revealed a progressive increase in MCP1 expression in the sciatic nerve of C57SOD1^G93A^ mice as the disease progresses (Figure S1F) by motor axons and Schwann cells (Figures S2J and S2K).

### The specific induction of MCP1 within the motor unit ameliorated the disease progression of C57SOD1^G93A^ mice

To hit the neuromuscular system of ALS mice, we selected the self-complementary adeno-associated virus serotype 9 (scAAV9) in light of its ability to target the spinal cord’s hijacking of the axonal transport machinery and travel along the nerve following intramuscular (i.m.) injection.^56^ A single bilateral i.m. injection of the scAAV9 expressing the enhanced *Green Fluorescent Protein* gene (scAAV9_GFP) under the cytomegalovirus (CMV) promoter efficiently transduced the motor unit in adult C57 and 129Sv mSOD1 mice, as demonstrated by the GFP expression in the skeletal muscles (Figures S3A–S3C), motor axons (Figures S3D–S3F), and spinal cord of scAAV9_GFP-treated mice (Figures S3G–S3J). Notwithstanding Benkhelifa-Ziyyat et al.^56^ showed that the scAAV9 i.m. injection in adult mice efficiently transduced both spinal MNs and astrocytes, we found that the transduction occurred specifically within MN perikarya without affecting the neighboring non-neuronal cells (Figures S3G and S3H). Although it is not possible to exclude the vector spreading in the circulation, our data suggest that gene transfer resulted mainly from retrograde axonal transport from the injected muscle to the related MN cell bodies.

**Figure 1:**
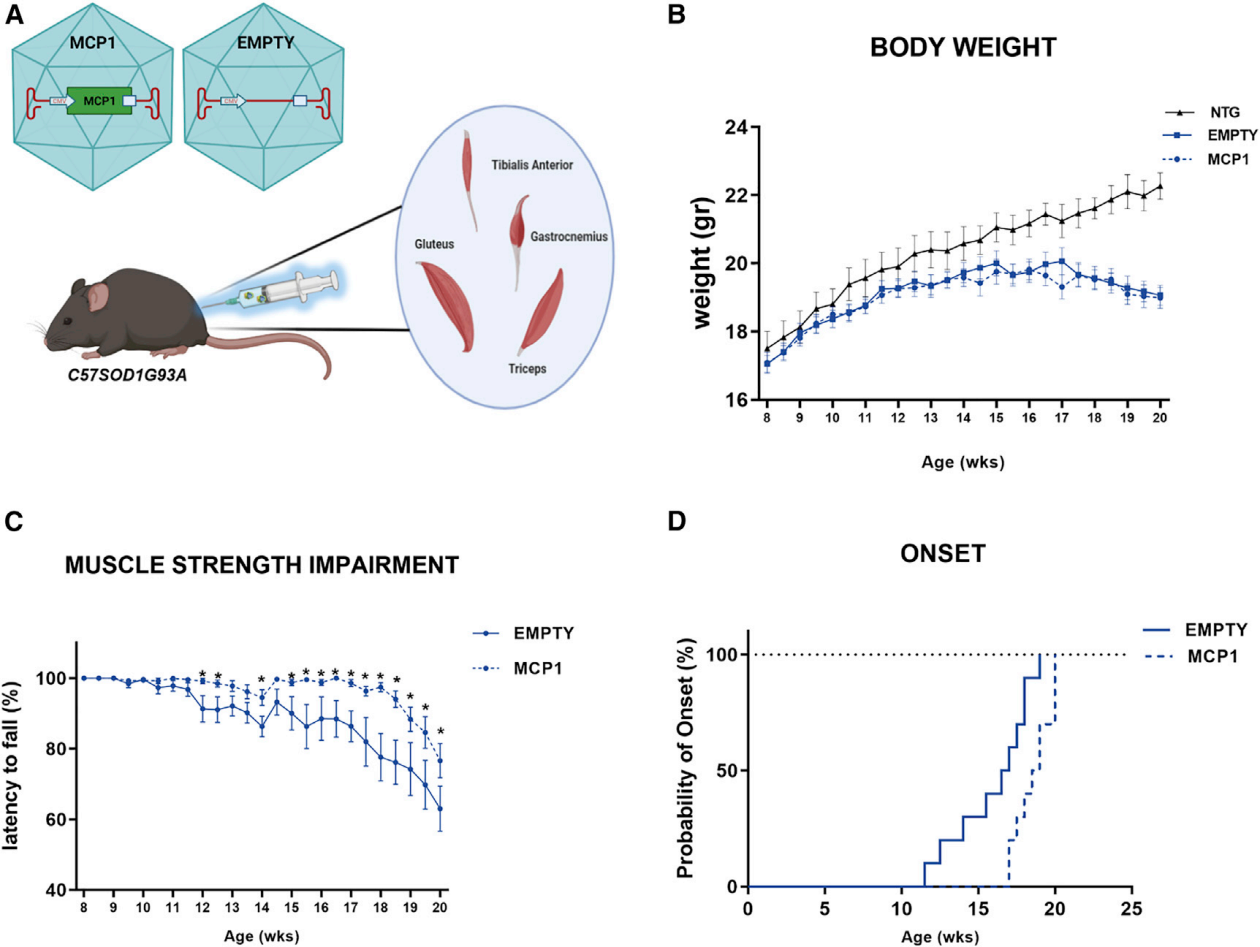
The scAAV9_MCP1 injection ameliorates the disease progression of C57SOD1^G93A^ mice (A) Experimental design. The engineered (scAAV9_MCP1) or empty (scAAV9(empty)) vector was administered in both hindlimb and forelimb muscles of 8-week-old C57SOD1^G93A^ mice (n = 18 per group). Image created in Biorender.com. (B and C) Recording of (B) body weight and (C) muscle strength impairment of scAAV9_MCP1- and scAAV9(empty)-treated mice until the symptomatic disease stage. Data are reported as means ± SEMs for each time point. ∗p < 0.05; by repeated-measures ANOVA with Sidak’s post-analysis. (D) scAAV9_MCP1-treated mice exhibit a postponement of the disease onset of approximately 2 weeks compared with the scAAV9(empty) group. Age of disease onset: empty 16 ± 0.8 weeks, MCP1 18.6 ± 0.4 weeks. Means ± SEMs. p = 0.0088 by Mantel-Cox log rank test.

To analyze the effect of the MCP1 boosting on the disease progression, the scAAV9 vector properly engineered with the murine sequence of *Mcp1* (scAAV9_MCP1) was injected in the skeletal muscles of the hindlimb (*gastrocnemius caput medialis*, GCM; *tibialis anterior*, TA; *gluteus maximus*, GM) and forelimb (*triceps brachii*, TB) of pre-symptomatic (8 weeks old) C57SOD1^G93A^ mice (Figure 1A). Given the early muscle deficit in mSOD1 mice,^57^^,^^58^ 8 mice per group were sacrificed approximately 2 weeks before the motor symptoms onset (14 weeks), while 10 mice per group were monitored until the symptomatic disease stage (20 weeks). A scAAV9(empty) vector was used as control (Figure 1A).

During the study, no difference in the body weight was observed between the two experimental groups of C57SOD1^G93A^ mice, excluding any major side effect upon the induction of a pro-inflammatory factor in ALS mice (Figure 1B). Notably, in the scAAV9_MCP1-treated mice, the impairment of muscle strength was delayed and progressed slowly up to 20 weeks of age (Figure 1C), leading to the postponement of the disease onset of approximately 2 weeks compared with the control group (empty 16 ± 0.8 weeks, MCP1 18.6 ± 0.4 weeks [means ± SEMs]) (Figure 1D).

### The MCP1 boosting in the hindlimb skeletal muscles of C57SOD1^G93A^ mice delayed the denervation atrophy and prompted muscle re-innervation

**Figure 2:**
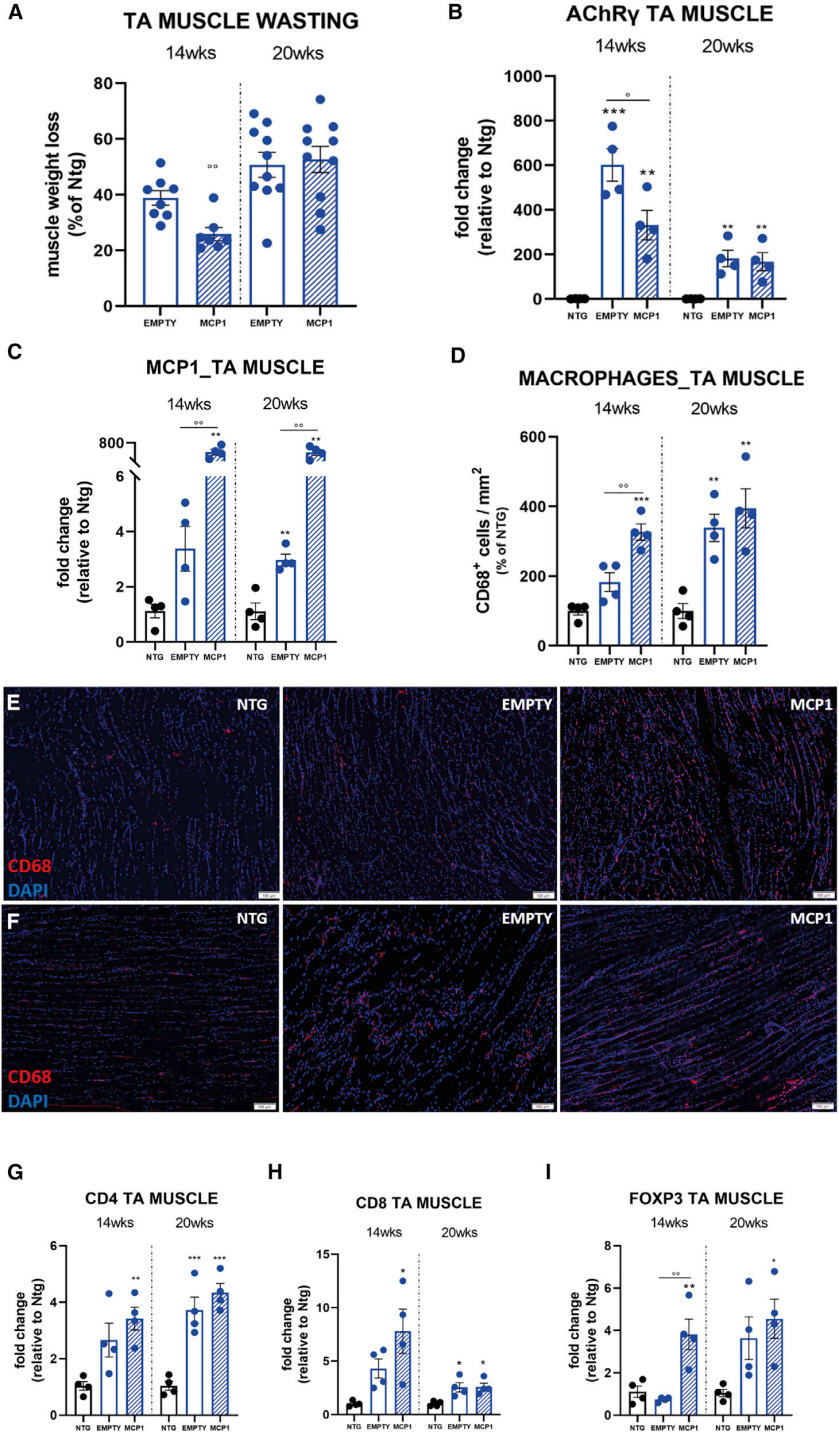
The scAAV9_MCP1 injection in C57SOD1^G93A^ mice delays the hindlimb muscle degeneration, promoting leukocyte recruitment (A) Muscle wasting was calculated by measuring the TA muscle weight of scAAV9_MCP1- and scAAV9(empty)-treated mice compared to relative Ntg littermates at each time point analyzed. The percentage of muscle atrophy was calculated relative to Ntg mice. Data are reported as means ± SEMs. n = 8 per group at 14 weeks; n = 10 per group at 20 weeks. (B and C) Real-time PCR analysis of (B) *AChRγ* and (C) *Mcp1* transcript in the TA muscle of scAAV9_MCP1- and scAAV9(empty)-treated mice compared to relative Ntg littermates. Data are normalized to β-actin and expressed as means ± SEMs. n = 4 per group at each time point. (D–F) Representative confocal micrographs of longitudinal sections of TA muscle of scAAV9_MCP1- and scAAV9(empty)-treated mice and Ntg littermates at (E) 14 and (F) 20 weeks stained with the phagocytic marker CD68 (red) and DAPI (nucleus, blue). Scale bar, 100 μm. (D) The relative quantifications demonstrate an increased macrophage infiltration in the TA muscle of scAAV9_MCP1-treated mice compared with the scAAV9(empty) group at 14 but not 20 weeks. Data are reported as means ± SEMs of 3–5 serial sections per muscle from n = 4 mice per experimental group at each time point. (G–I) Real-time PCR analysis of (G) *CD4*, (H) *CD8a*, and (I) *Foxp3* transcript in the TA muscle of scAAV9_MCP1- and scAAV9(empty)-treated mice compared to relative Ntg littermates at 14 and 20 weeks. Data are normalized to β-actin and expressed as means ± SEMs. n = 4 per experimental group at each time point. ∗p < 0.05, ∗∗p < 0.01, ∗∗∗p < 0.001 Ntg versus empty or MCP1; °p < 0.05, °°p < 0.01 empty versys MCP1 by 1-way ANOVA with Fisher post-analysis.

The impairment of skeletal muscles is an early event in the ALS pathogenic cascade,^59^, ^60^, ^61^ pivotal in determining the motor ability of mSOD1 mice. To dissect the effect of the MCP1 boosting on muscular degeneration, we investigated the TA muscle given the advanced susceptibility of the lower motor units in mSOD1 mice^62^ and the high composition in fast-fatigable fibers, which are early affected by the disease.^57^^,^^63^ In keeping with the ameliorated clinical phenotype, at 14 weeks, the TA muscle of scAAV9_MCP1-treated mice was less jeopardized (Figure 2A). Our analysis recorded a reduction of 38.8% ± 2.6% (mean ± SEM) of the muscle mass in the scAAV9(empty)-treated mice compared with non-transgenic (Ntg) littermates, which decreased to 25.9% ± 2.3% (mean ± SEM) upon scAAV9_MCP1 injection. Moreover, a significant downregulation of the fetal gamma-subunit of the acetylcholine receptor (*AChRγ*) was recorded upon MCP1 induction, indicating considerable preservation of muscle innervation compared with the scAAV9(empty) group^64^ (Figure 2B). Accordingly, the histological analysis revealed a reduced percentage of denervated neuromuscular junctions (NMJs) in the hind paw muscle of scAAV9_MCP1- compared with the scAAV9(empty)-treated mice (Figures S4A and S4C). Intriguingly, the histological examination uncovered a higher proportion of motor axons expressing the Growth Associated Protein 43 (GAP43) upon MCP1 boosting, indicating the reinstatement of the nerve sprouting in the fast-twitch muscle of SOD1^G93A^ mice^65^^,^^66^ (Figures S4A and S4D). A higher rate of GAP43^+^ regenerating motor axons and innervated NMJs was still detectable in the scAAV9_MCP1-treated group at the symptomatic disease stage (Figures S4B–S4D), albeit without impinging on the *AChRγ* transcription level or TA muscle atrophy compared with the control group (Figures 2A and 2B).

### The early MCP1 boosting favored the establishment of an anti-inflammatory milieu in the skeletal muscles of C57SOD1^G93A^ mice

A great deal of evidence correlates the protective effect of the MCP1-mediated inflammation to its chemoattractant activity toward immune cells, particularly macrophages,^29^^,^^49^^,^^67^ which are pivotal at sustaining skeletal muscle healing upon damage.^68^ Accordingly, we next analyzed the chemokine levels within the TA muscle of the scAAV9_MCP1-treated and control group at the pre-symptomatic and symptomatic disease stages. *Mcp1* transcript was significantly upregulated in the TA muscle of C57SOD1^G93A^ mice compared with the Ntg littermates at 20 but not 14 weeks of age and dramatically increased by scAAV9_MCP1 injection at both time points (Figure 2C). Notably, the extent of chemokine induction recorded in scAAV9_MCP1-treated mice at 14 and 20 weeks of age was similar, confirming the ability of the scAAV9 at inducing the chemokine several weeks after the single i.m. injection (mRNA FC versus Ntg 14 weeks: 548.8 ± 81.7; 20 weeks: 542.1 ± 82.5 [means ± SEMs]). Accordingly, we found a significant increase in the recruitment of phagocytic CD68^+^ macrophages within the TA muscle of scAAV9_MCP1-treated mice compared with the control groups. This effect was significant at 14 but not 20 weeks of age, when macrophages massively infiltrate the skeletal muscle of symptomatic mSOD1 mice^69^ (Figures 2D–2F).

CCR2 is also expressed by activated T lymphocytes,^70^ instrumental in the regenerative mechanisms of skeletal muscles.^52^ Although an increasing trend in *CD4* and *CD8a* transcript levels was recorded upon MCP1 boosting, *FoxP3* mRNA was significantly upregulated at 14 weeks, suggesting an increased infiltration of T regulatory lymphocytes (Tregs) compared with the scAAV9(empty) group (Figures 2G–2I). As indicated by the fine kinetic governing the immune response within the injured muscle,^53^^,^^71^^,^^72^ T regs are the last immune cells infiltrating into the injured tissue pivotal at sustaining the pro-healing program.^73^^,^^74^ In keeping with this, a reduced release of the elastase enzyme was recorded in the TA muscle of scAAV9_MCP1-treated mice compared with the control group at 14 weeks (Figures S5A and S5B), indicating that neutrophils, which are the first immune cells recruited by the chemotactic gradient established within the damaged muscle,^71^^,^^75^^,^^76^ have already given way to leukocytes.

**Figure 3:**
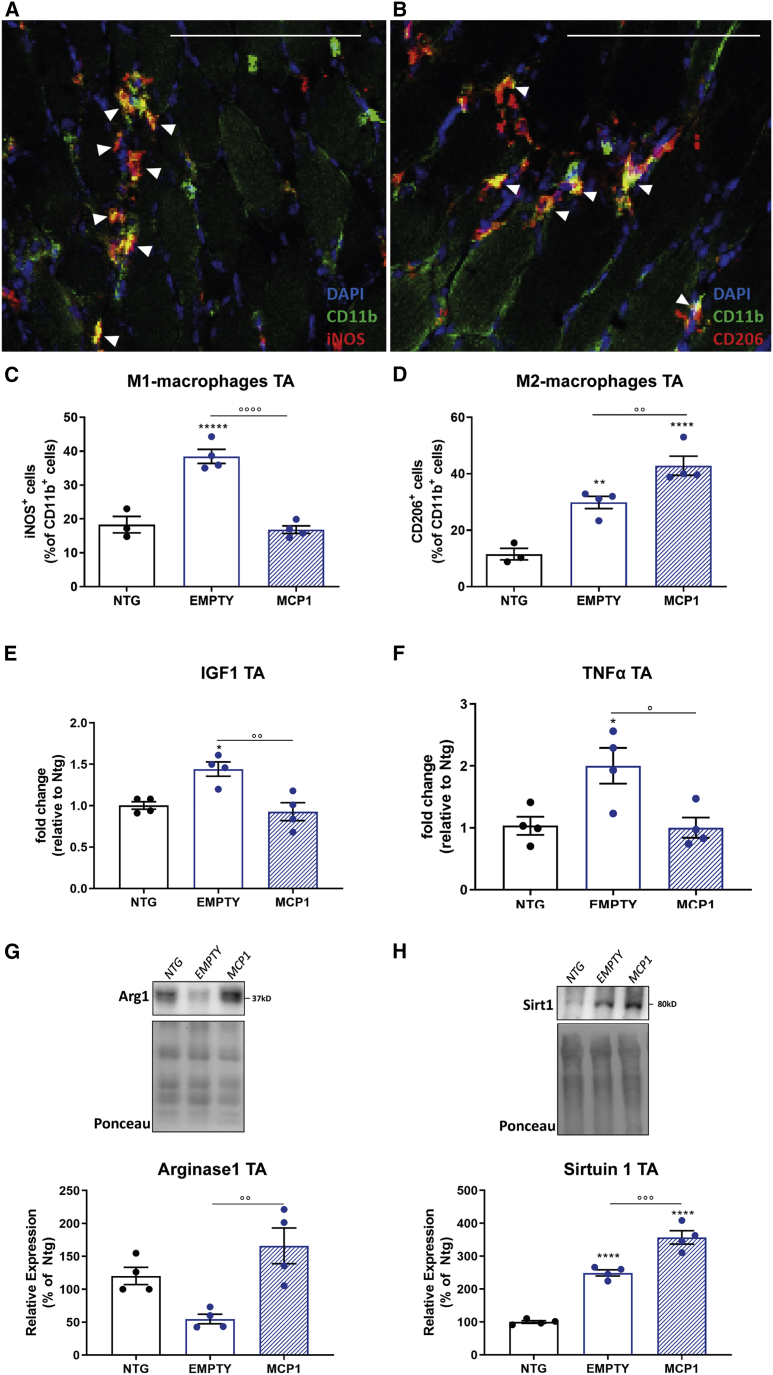
The MCP1 boosting enables the macrophage phenotypic switch in the hindlimb muscles of C57SOD1^G93A^ mice (A–D) Representative confocal micrographs of coronal sections of TA muscle of scAAV9_MCP1- and scAAV9(empty)-treated mice and Ntg littermates at 14 weeks stained with the myeloid marker CD11b (green). (A) The pro-inflammatory iNOS (red) or (B) anti-inflammatory CD206 (red) marker and DAPI (nucleus, blue). White arrowheads indicate CD11b^+^CD206^+^ or CD11b^+^iNOS^+^ macrophages. Scale bar, 100 μm. The relative quantifications show a decreasing percentage of (C) M1 pro-inflammatory and a compensatory increase of (D) M2 anti-inflammatory macrophages in scAAV9_MCP1- compared with scAAV9(empty)-treated mice. Data are reported as means ± SEMs of 3–5 serial sections per muscle from n = 3 Ntg and n = 4 SOD1^G93A^ mice per group. (E and F) Real-time PCR analysis of (E) *Igf1* and (F) *Tnf-α* in the TA muscle of scAAV9_MCP1- compared with scAAV9(empty)-treated mice at 14 weeks. Data are normalized to β-actin and expressed as fold change relative to the Ntg mice. n = 4 per group. (G and H) Representative immunoblot images and relative quantifications of (G) arginase 1 and (H) sirtuin 1 expression in the TA muscle of scAAV9_MCP1- and scAAV9 (empty)-treated mice and Ntg littermates at 14 weeks. n = 4 per experimental group. Data are reported as means ± SEMs. ∗p < 0.05, ∗∗p < 0.01, ∗∗∗∗p < 0.0001 Ntg versus empty or MCP1; °p < 0.05, °°p < 0.01, p < 0.001, °°°°p < 0.0001 empty versus MCP1 by 1-way ANOVA with Fisher post-analysis.

We next investigated the inflammatory fingerprint acquired by the MCP1-recruited immune cells in the skeletal muscle of 14-week-old C57SOD1^G93A^ mice. To this end, we assessed the expression level of the inducible nitric oxide synthase (iNOS) and mannose receptor (CD206), markers of classically (M1) pro-inflammatory and alternatively (M2) anti-inflammatory activated macrophages, respectively^77^ (Table S1). The histological analysis revealed that the percentage of the M1 iNOS^+^ myeloid cells infiltrated in the TA muscle significantly dropped upon MCP1 boosting, whereas a remarkably increase in the M2-CD206^+^ counterpart was recorded compared with the scAAV9(empty)-treated mice (Figures 3A–3D). The examination of the muscular inflammatory milieu in the scAAV9_MCP1-treated mice revealed a significant downregulation of the insulin-like growth factor 1 (*Igf1*) compared with the control group (Figure 3E), a cytokine released by M1-macrophages exerting an autocrine function pivotal to trigger the M2-gene program.^78^ In addition, the significant downregulation of the pro-inflammatory cytokine tumor necrosis factor α (*Tnf*-α) (Figure 3F) and increased expression of the anti-inflammatory factor arginase 1 (Arg1) (Figure 3G) suggested the establishment of an anti-inflammatory environment in the hind paw muscle of C57SOD1^G93A^ mice 6 weeks after the scAAV9_MCP1 injection. In keeping with this, in the TA muscle of scAAV9_MCP1-treated mice, we found a higher increase in Sirtuin 1 (Sirt1) deacetylase protein level (Figure 3H), whose overexpression in skeletal muscle is associated with a macrophage polarization shift toward the anti-inflammatory phenotype^79^^,^^80^ (Table S1).

At 20 weeks of age, despite the significant *Mcp1* upregulation (Figure 2C), no difference in macrophage (Figures 2D and 2F) and T cell (Figures 2G–2I) recruitment was found within the TA muscle of scAAV9_MCP1- and scAAV9(empty)-treated mice. In addition, no variation was registered in the inflammatory response between the two experimental groups of mSOD1 mice other than a significant reduction in *Igf1* levels (Figure S5C) and expression of the pro-inflammatory factor cytochrome b-245 heavy chain (gp91^PHOX^) in the scAAV9_MCP1-treated group (Figures S5D and S5E; Table S1).

### The early MCP1-mediate boosting of the immune response triggered myogenic progenitor cell differentiation in the hindlimb skeletal muscle of C57 SOD1^G93A^ mice

The ability of recruited immune cells to govern the myogenic program upon damage is dependent strictly on the acquired inflammatory fingerprint. While the M1-macrophages promote activation and proliferation of the myogenic progenitors, their switch toward the M2 phenotype is fundamental to sustain the final commitment of satellite cells (SCs) toward myogenesis.^71^^,^^81^, ^82^, ^83^, ^84^

**Figure 4:**
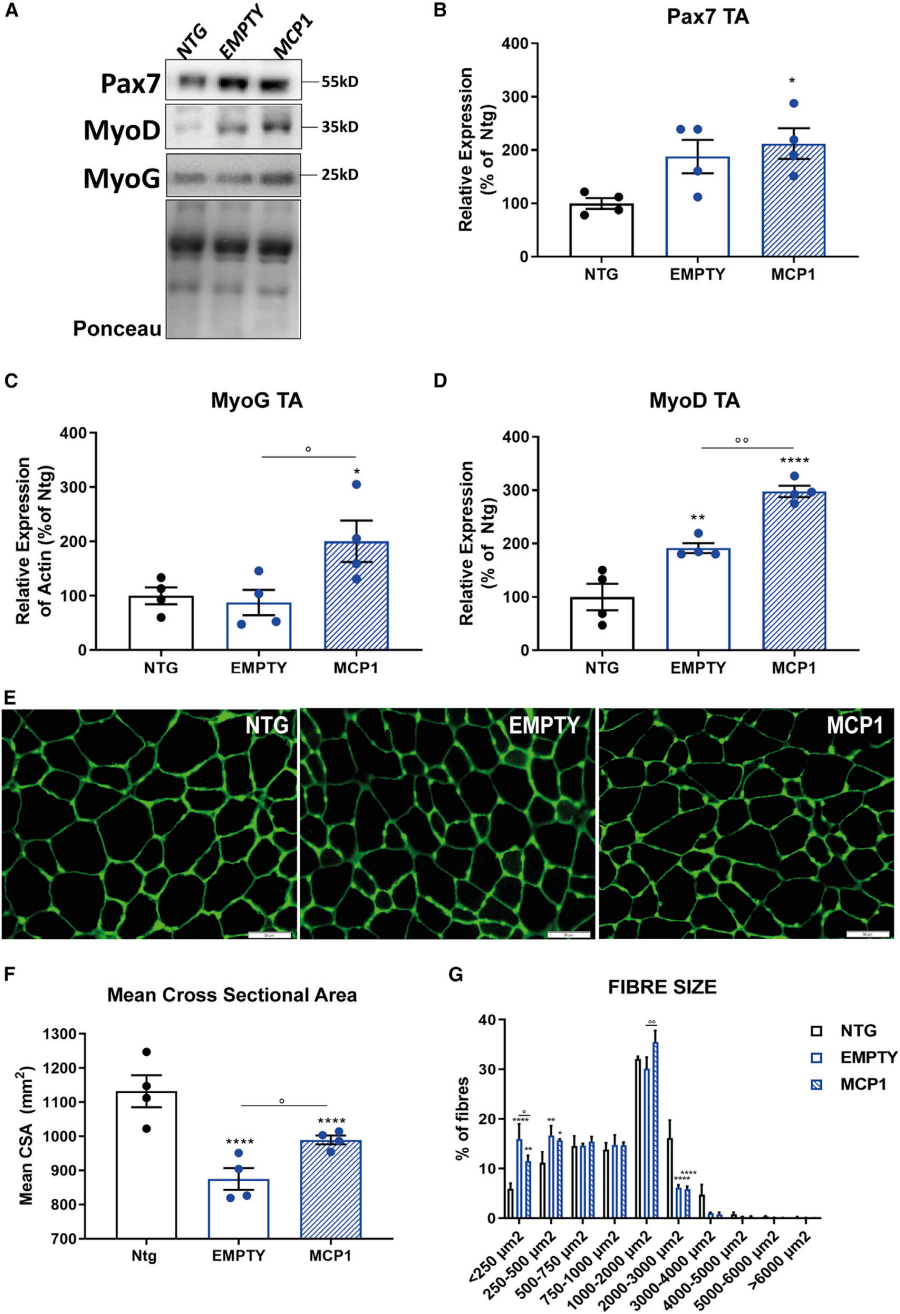
The MCP1-mediated immune response triggers the myogenic program in the hindlimb muscles of C57SOD1^G93A^ mice (A–D) Representative immunoblot images and relative densitometric analysis of (A and B) Pax7, (A and C) MyoG, and (A and D) MyoD expression in TA muscle extracts of scAAV9_MCP1- and scAAV9(empty)-treated mice and Ntg littermates at 14 weeks. n = 4 per experimental group. (E) Representative confocal micrograph of coronal sections of the TA muscle of scAAV9_MCP1- and scAAV9(empty)-treated mice and Ntg littermates at 14 weeks stained with laminin (green). Scale bar, 50 μm. (F and G) Quantitative analysis of (F) the cross-sectional area (CSA) mean of TA muscle fibers and (G) the size distribution at 14 weeks. Data are reported as means ± SEMs of 3–5 serial sections per muscle from n = 4 mice per group. ∗p < 0.05, ∗∗p < 0.01, ∗∗∗∗p < 0.0001 Ntg versus empty or MCP1; °p < 0.05, °°p < 0.01, empty versus MCP1 by (B–D, and F) 1-way or (G) 2-way ANOVA with Fisher post-analysis.

We showed that scAAV9_MCP1 injection favored the establishment of an anti-inflammatory pro-regenerative milieu in the TA muscle of C57SOD1^G93A^ mice. Therefore, we next assessed the impact of MCP1 boosting on the expression of two critical myogenic factors in the TA muscle of 14-week-old C57SOD1^G93A^ mice: Paired Box 7 (Pax7), the hallmark of SCs stemness,^85^ and Myogenin (MyoG), a marker of early commitment and differentiation.^86^ MyoG, but not Pax7, was significantly upregulated in the TA muscle of scAAV9_MCP1-treated mice compared with the control group (Figures 4A–4C). In addition, our analysis revealed that the expression of the Myoblast Determination protein 1 (MyoD), a transcription factor critical in defining the fate of the activated SCs,^87^^,^^88^ was significantly increased upon MCP1 boosting (Figures 4A and 4D). Suitably, the histological examination showed a reduction in the percentage of quiescent (Pax7^+^/MyoD^−^) and a significant increase in differentiating (Pax7^−^/MyoD^+^) SCs in the TA muscle of scAAV9_MCP1-treated mice compared with controls (Figures S6A and S6D). According to the increased myogenic activity, a higher percentage of centralized myonuclei was recorded in the hind paw muscle of C57SOD1^G93A^ mice upon MCP1 boosting (percentage versus Ntg: 191.0 ± 54.7, empty; 380.7 ± 44.2, MCP1 [means ± SEMs]), indicating an intense regenerative process compared with the scAAV9(empty) group^89^ (Figures S6B and S6E).

Given that the shift from large fast-twitch to small slow-twitch fibers is a common hallmark of ALS,^90^^,^^91^ we next estimated the TA oxidative muscle fiber composition by the succinate dehydrogenase (SDH) histochemical assay. As expected, SOD1^G93A^ mice exhibited a higher percentage of slow-twitch oxidative fibers (87.3% ± 1.4%, mean ± SEM) compared with Ntg littermates (52.9% ± 0.1%, mean ± SEM), which significantly decreased upon MCP1 boosting (74.7% ± 1.9%, mean ± SEM) (Figures S6C and S6F). In keeping with this, the mean cross-sectional area (CSA) of the TA muscle fibers was higher in the scAAV9_MCP1-treated mice compared with the control group (Figures 4E and 4F). In addition, the histological analysis revealed a significant reduction in the percentage of the small (<205 μm^2^) and compensatory preservation of medium (1,000–2,000 μm^2^) fibers in the TA muscle of C57SOD1^G93A^ mice upon MCP1 boosting (Figures 4E and 4G).

Altogether, this evidence suggests a possible correlation between the M2 polarization of the muscular inflammatory milieu and the enhanced myogenic activity in the TA muscle of the scAAV9_MCP1-treated mice. This effect progressively weakened during the disease progression as, at 20 weeks, we did not record any differences between the 2 groups of SOD1^G93A^ mice in the myogenic program, despite the significant *Mcp1* upregulation in the skeletal muscle (data not shown).

### The MCP1-mediated boosting preserved motor axons from degeneration in the sciatic nerves of C57SOD1^G93A^ mice

We previously reported an association between the activation of the MCP1-mediated pathway in the PNS and a slower disease progression of SOD1^G93A^ mice.^26^ Therefore, we analyzed the effect of chemokine induction and the eventual immune cell recruitment within the sciatic nerve of C57SOD1^G93A^ scAAV9_MCP1 i.m.-injected mice. Unlike skeletal muscle, the treatment modestly increased *Mcp1* levels in the sciatic nerves at 14 weeks (Figure S7A). However, this did not further enhance the recruitment of the macrophages nor the cytotoxic T cells, as demonstrated by the unchanged levels of *CD68* and *CD8a* transcripts than the scAAV9(empty)-treated group (Figures S7B and S7C). In keeping with this, no difference in the *Tnf-α* transcription was recorded in the sciatic nerve of SOD1^G93A^ mice compared with the Ntg littermates (Figure S7D).

At the symptomatic disease stage, the gene expression analysis showed a significant and similar upregulation of *Mcp1* and *CD68* transcripts in the sciatic nerves of both groups of C57SOD1^G93A^ mice compared with Ntg littermates (Figures S7A and S7B). Intriguingly, our investigation revealed a significant decrease in the recruitment of the CD68^+^ macrophages (Figure S7B) and a reduction trend in the infiltration of the CD8^+^ T lymphocytes (Figure S7C) along motor axons of C57SOD1^G93A^ mice upon MCP1 boosting. The diminished leukocyte recall significantly abated the PNS inflammation in scAAV9_MCP1-treated mice, as demonstrated by the *Tnf-α* downregulation compared with the control group (Figure S7D). This effect was associated with an increased expression of the p75-neurotrophin receptor (p75^NTR^) within the sciatic nerves of scAAV9_MCP1-treated mice compared with controls (Figures S7E and S7F). Although the role of p75^NTR^ within peripheral nerves is still controversial,^92^ recent evidence correlates the reduced neurotrophin receptor expression level in mSOD1 mice upon a nerve crush with a poor ability to prepare for regeneration and remyelination, highlighting its involvement in mediating motor axon recovery and plasticity.^17^^,^^93^ Suitably, while the heavy neurofilament (NF200) and myelin basic protein (MBP) levels were significantly downregulated in the sciatic nerves of symptomatic SOD1^G93A^ mice, their expression was unchanged in the scAAV9_MCP1-treated mice compared with the Ntg littermates (Figures S7E, S7G, and S7H). Altogether, these observations suggest that the early scAAV9_MCP1 i.m. injection leads to a significant PNS preservation in C57SOD1^G93A^ mice at the advanced disease stage.

### The MCP1 induction within spinal motor neurons of C57SOD1^G93A^ mice is protective by decreasing neuroinflammation

We showed that the scAAV9_GFP spreads retrogradely from the injected muscles alongside the motor unit of mSOD1 mice, finally transducing MN soma (Figure S3). Therefore, we analyzed the effect of chemokine induction on the neurodegenerative signature of ALS.

**Figure 5:**
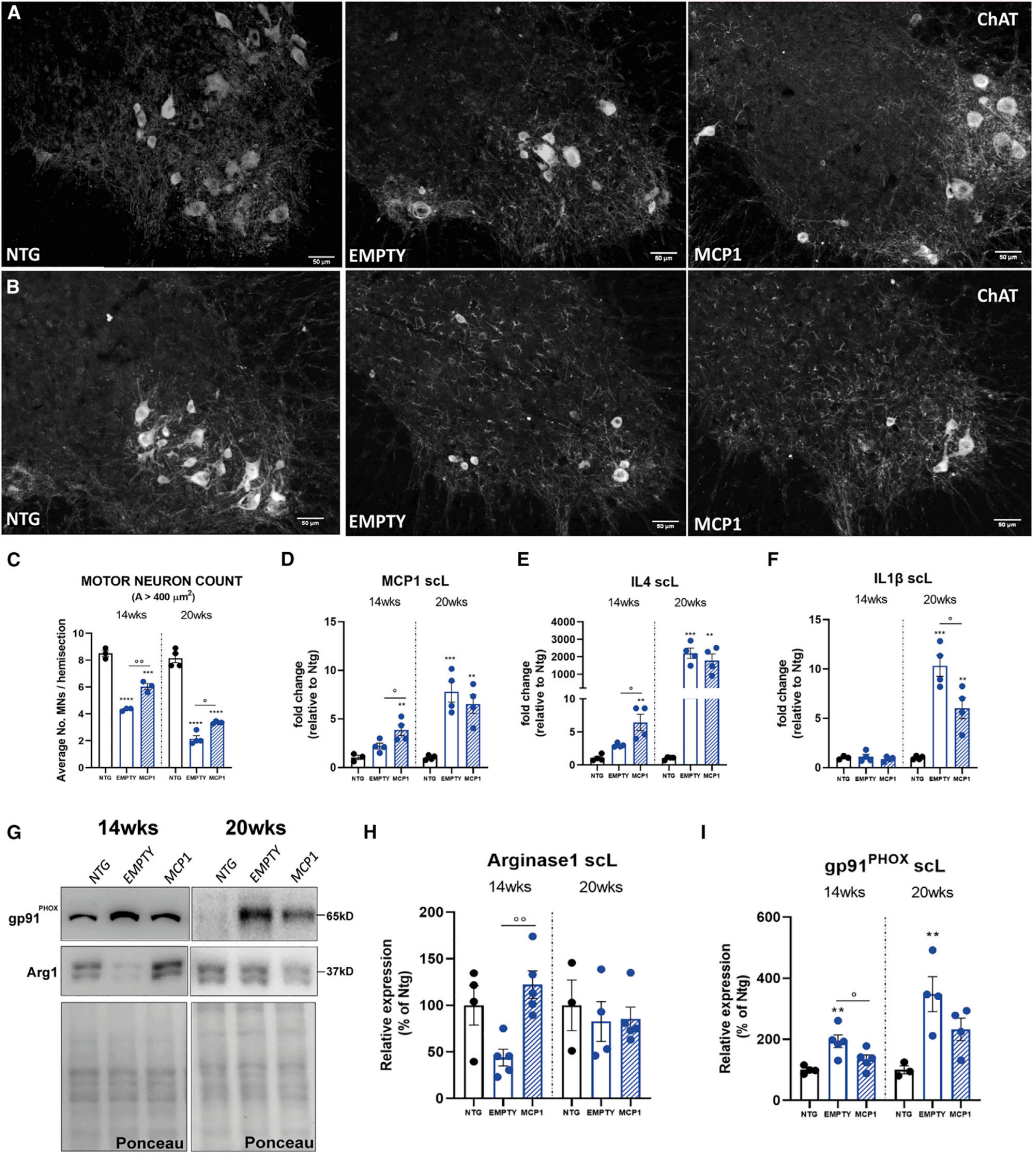
The MCP1 induction prevents motoneuron degeneration and modulates neuroinflammation in the CNS of C57SOD1^G93A^ mice (A and B) Representative ChAT-stained coronal sections of the lumbar spinal cord of scAAV9_MCP1- and scAAV9(empty)-treated mice and Ntg littermates at (A) 14 weeks and (B) 20 weeks. Scale bar, 50 μm. (C) Motor neuron counts. Data are expressed as means ± SEMs of MNs (area ≥400 μm^2^) counted per section. The quantitative analysis was performed on 12 serial ChAT-stained sections of the L3–L5 spinal cord segment; 14 weeks: n = 3 per group; 20 weeks: n = 4 per group. (D–F) Real-time PCR analysis of (D) *Mcp1*, (E) *Il4*, and (F) *Il1β* transcript in the lumbar spinal cord of scAAV9_MCP1- and scAAV9(empty)-treated mice compared to relative Ntg littermates at 14 and 20 weeks. Data are normalized to β-actin and expressed as means ± SEMs; n = 4 per experimental group. (G–I) Representative immunoblot images and relative densitometric analysis of (G and H) arginase 1 and (G and I) gp91^PHOX^ expression in lumbar spinal cord extracts of scAAV9_MCP1- and scAAV9(empty)-treated mice and Ntg littermates at 14 and 20 weeks. Data are reported as means ± SEMs; 14 weeks: n = 4 per experimental group; 20 weeks: n = 3 Ntg and n = 4 SOD1^G93A^ mice per group. ∗∗p < 0.01, ∗∗∗p < 0.001, ∗∗∗∗p < 0.0001 Ntg versus empty or MCP1; °p < 0.05, °°p < 0.01 empty versus MCP1 by 1-way ANOVA with Fisher post-analysis.

The gene expression analysis confirmed a significant *Mcp1* upregulation in the lumbar spinal cord of scAAV9_MCP1 i.m.-treated mice compared with the control groups at 14 weeks (Figure 5D). Conversely, at 20 weeks, no difference in the chemokine transcription was recorded between the 2 groups of C57SOD1^G93A^ mice (Figure 5D), suggesting that, at the full-blown stage, the massive MCP1 expression by microglia^94^^,^^95^ (Figures S1E, S2C, and S2F) may mask the neuronal scAAV9-mediated induction.

Intriguingly, upon chemokine boosting, lumbar MNs were significantly spared from the degenerative phenomenon, even several weeks from the scAAV9_MCP1 injection (MN number at 14 weeks: 4.3 ± 0.06, empty, 5.9 ± 0.29 MCP1; 20 weeks: 2.1 ± 0.22 empty, 3.3 ± 0.04 MCP1 [mean ± SEM]) (Figures 5A–5C).

At 14 weeks, the anti-inflammatory markers interleukin-4 (*Il4)* (Figure 5E) and Arg1 (Figures 5G and 5H) were significantly upregulated, whereas the expression of the pro-inflammatory factor gp91^PHOX^ (Figures 5G and 5I) was decreased in the CNS of scAAV9_MCP1-treated mice. Conversely, at the symptomatic disease stage, the treatment resulted in a significant downregulation of the pro-inflammatory factor *Il1β* (Figure 5F) and a reduction trend in the gp91^PHOX^ expression (Figures 5G and 5I) compared with the scAAV9(empty) group (Table S1). These modifications did not alter the glia activation state, as demonstrated by the unchanged expression levels of the ionized calcium-binding adapter molecule 1 (Iba1) and glial fibrillary acidic protein (GFAP) between the 2 groups of C57SOD1^G93A^ mice (Figures S8A and S8B). These data suggest that the MCP1 boosting in spinal MNs may have extended the so-called stable phase of the disease in SOD1^G93A^ mice,^96^^,^^97^ preserving the glia toward an anti-inflammatory phenotype, followed later by the inhibition of the pro-inflammatory environment, which is reflected in MN preservation.

### The MCP1 boosting in forelimb skeletal muscles of C57 SOD1^G93A^ mice delayed the denervation atrophy through immune-related myogenesis

The mSOD1 mice first develop hindlimb tremors and then progressive hindlimb weakness with rapidly deteriorating gait, eventually culminating in the paralysis of one or both hindlimbs.^62^^,^^98^, ^99^, ^100^ Forelimb function comparatively is spared throughout the disease progression, indicating a distinct susceptibility of the forepaw motor units in mSOD1 mice.^62^^,^^101^^,^^102^ This evidence highlighted the importance of the contribution of the forelimbs in the disease progression of ALS mice, particularly at the advanced disease stage.^102^

**Figure 6:**
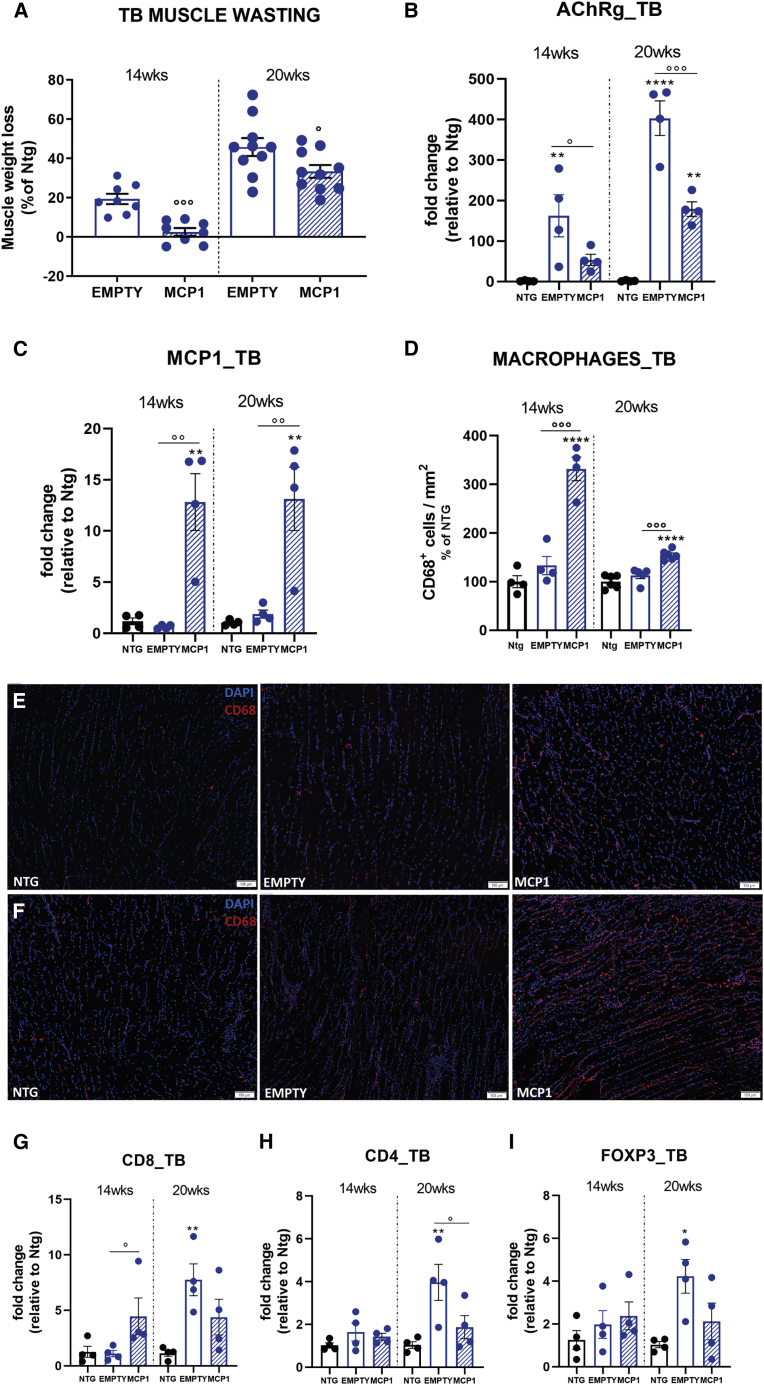
The scAAV9_MCP1 injection reduces the forelimb muscle degeneration, promoting leukocyte recruitment in C57SOD1^G93A^ mice (A) Muscle wasting was calculated by measuring the TB muscle weight of scAAV9_MCP1- and scAAV9(empty)-treated mice compared to relative Ntg littermates at each time point analyzed. The percentage of muscle atrophy was calculated relative to Ntg mice. Data are reported as means ± SEMs; 14 weeks: n = 8 per group; 20 weeks: n = 10 per group. (B and C) The real-time PCR analysis of (B) *AChRγ* and (C) *Mcp1* transcript in the TB muscle of scAAV9_MCP1- and scAAV9(empty)-treated mice compared to relative Ntg littermates at 14 and 20 weeks. Data are normalized to β-actin and expressed as means ± SEMs; n = 4 per experimental group at each time point analyzed. (D–F) Quantification and representative confocal micrograph of longitudinal sections of TB muscle of scAAV9_MCP1- and scAAV9(empty)-treated mice and Ntg littermates at (D and E) 14 and (D and F) 20 weeks stained with the phagocytic marker CD68 (red) and DAPI (nucleus, blue). Scale bar, 100 μm. Data are reported as means ± SEMs of 3–5 serial sections per muscle from n = 4 mice per group at 14 weeks and n = 6 mice per group at 20 weeks. (G–I) Real-time PCR analysis of (G) *CD8a*, (H) *CD4*, and (I) *Foxp3* transcript in the TB muscle of scAAV9_MCP1- and scAAV9(empty)-treated mice compared to relative Ntg littermates at 14 and 20 weeks. Data are normalized to β-actin and expressed as means ± SEMs; n = 4 per experimental group at each time point. ∗p < 0.05, ∗∗p < 0.01, ∗∗∗∗p < 0.0001 Ntg versus empty or MCP1; °p < 0.05, °°p < 0.01, °°°p < 0.001 empty versus MCP1 by 1-way ANOVA with Fisher post-analysis.

Likewise the hindlimbs, alterations in the forepaws could be detectable before evident motor impairment.^58^ Accordingly, at 14 weeks, the TB muscle of SOD1^G93A^ mice has already lost the 19.3% ± 2.6% (mean ± SEM) of its mass compared with the Ntg littermates, which increased to 45.8% ± 4.6% (mean ± SEM) at 20 weeks. Notably, the MCP1 boosting significantly preserved the forepaw muscle of C57SOD1^G93A^ mice against the atrophic phenomenon reducing the muscle mass loss to 2.5% ± 1.9% and 33.3% ± 3.2%, respectively (means ± SEMs) (Figure 6A). Suitably, starting from 14 weeks, our analysis showed a significant *AChRγ* upregulation in the TB muscle of SOD1^G93A^ mice, an effect magnified at 20 weeks, corroborating the early and progressive NMJ alteration before the appearance of any sign of motor impairment. Notably, the MCP1 boosting remarkably prevented the NMJ denervation as demonstrated by the significant *AChRγ* downregulation compared with the scAAV9(empty)-treated mice at both time points (Figure 6B).

As for the TA muscle, a single scAAV9_MCP1 i.m. injection resulted in a long-lasting *Mcp1* upregulation compared with the control groups and with the same extent at both the considered time points (Figure 6C). Suitably, macrophage recruitment dramatically increased in the TB muscle of scAAV9_MCP1-treated mice compared with the control groups at 14 and 20 weeks (Figures 6D–6F). In addition, MCP1 boosting fostered the infiltration of cytotoxic CD8^+^ T cells, but not CD4^+^ T lymphocytes and FoxP3^+^ Tregs, only at 14 weeks, suggesting an early inflammatory response within the forepaw muscle. Indeed, in the scAAV9(empty)-treated mice, a slightly heightened level of T cell and Treg infiltration was recorded only at the symptomatic disease stage (Figures 6G–6I).

**Figure 7:**
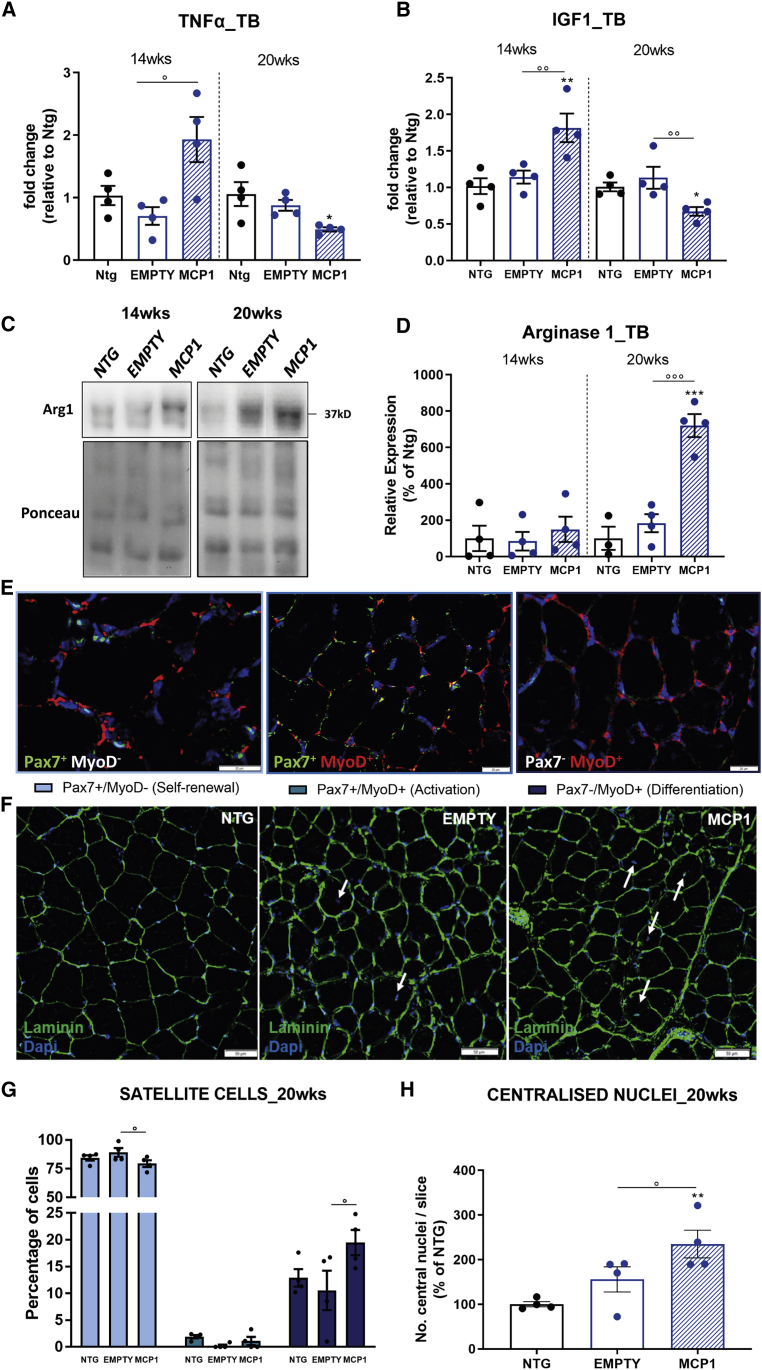
The MCP1-mediated immune response triggers the myogenic program in the forelimb muscles of C57SOD1^G93A^ mice (A and B) Real-time PCR analysis of (A) *Tnf-α* and (B) *Igf1* transcript in the TB muscle of scAAV9_MCP1- and scAAV9(empty)-treated mice compared to relative Ntg littermates at 14 and 20 weeks. Data are normalized to β-actin and expressed as means ± SEMs; n = 4 per experimental group. (C and D) Representative immunoblot images and relative densitometric analysis of arginase 1 expression in TB muscle extracts of scAAV9_MCP1- and scAAV9(empty)-treated mice and Ntg littermates at 14 and 20 weeks. Data are reported as means ± SEMs; n = 4 per group at each time point. (E and F) Confocal micrographs of coronal sections of TB muscle stained with (E) Pax7 (green), MyoD (red), and DAPI (blue) or (F) laminin (green) and DAPI (blue) of symptomatic scAAV9_MCP1- and scAAV9(empty)-treated mice and Ntg littermates. Scale bars, 20 μm (E); 50 μm (F). (G) Analysis of satellite cells’ dynamic in the TB muscle of scAAV9_MCP1-treated mice compared with the scAAV9(empty) group at 20 weeks. (H) Morphometric analysis of centralized myonuclei in the TB muscle of scAAV9_MCP1-treated mice compared with the scAAV9(empty) group at 20 weeks. Data are reported as means ± SEMs of 3–5 serial sections per muscle from n = 4 mice per experimental group. ∗p < 0.05, ∗∗p < 0.01, ∗∗∗p < 0.001 Ntg versus MCP1; °p < 0.05, °°p < 0.01, °°°p < 0.001 empty versus MCP1 by (A, B, D, and H) 1-way ANOVA or (G) 2-way ANOVA with Fisher post-analysis.

This evidence was corroborated by the *Tnf-α* upregulation recorded in the TB muscle of 14-week-old scAAV9_MCP1-treated mice compared with controls (Figure 7A) and by the unchanged expression of Arg1 between the 2 groups of SOD1^G93A^ mice (Figures 7C and 7D), suggesting a massive infiltration of M1-polarized leukocytes 6 weeks after the scAAV9_MCP1 injection. Notably, our analysis revealed a significant increase in *Igf1* transcript in the TB muscle of scAAV9_MCP1-treated mice (Figure 7B), indicating the ongoing switching of infiltrated M1 cells toward the M2 pro-healing phenotype.^78^

Accordingly, at 20 weeks, the *Tnf-α* downregulation (Figure 7A) heightened Arg1 expression and decreased *Igf1* transcription compared with controls (Figures 7B–7D) suggested the establishment of an anti-inflammatory muscular milieu 12 weeks after the scAAV9_MCP1 injection.

The histological analysis of transverse TB muscle sections showed that the MCP1-mediated immune cell infiltration did not significantly modify the quiescent status of the SCs at the pre-symptomatic disease stage (Figures S9A and S9C). Accordingly, no significant difference in the percentage of centralized myonuclei was recorded between the 2 groups of SOD1^G93A^ mice at 14 weeks, albeit an increasing trend was noticeable upon MCP1 boosting (Figures S9B and S9D). Conversely, at 20 weeks, the switch of the recruited leukocytes toward the M2 pro-healing phenotype promoted the TB regeneration in scAAV9_MCP1-treated mice, as demonstrated by the increased percentage of differentiating Pax7^−^/MyoD^+^ SCs (empty: 10.5% ± 3.6%; MCP1: 19.5% ± 2.3% [means ± SEMs]) (Figures 7E–7G) and centralized myonuclei (percentage versus Ntg: 155.6 ± 28.2, empty; 234.8 ± 31.8, MCP1 [means ± SEMs]) compared with the control group (Figures 7F and 7H).

### The MCP1 induction in 129SvSOD1^G93A^ mice exacerbated inflammation in the periphery, worsening the clinical phenotype

The data collected demonstrated the beneficial action of MCP1 boosting within the motor unit of C57SOD1^G93A^ mice. Therefore, we assessed whether the chemokine induction in 129SvSOD1^G93A^ mice, which show a faint activation of the MCP1 axis, was able to ameliorate the disease progression.

**Figure 8:**
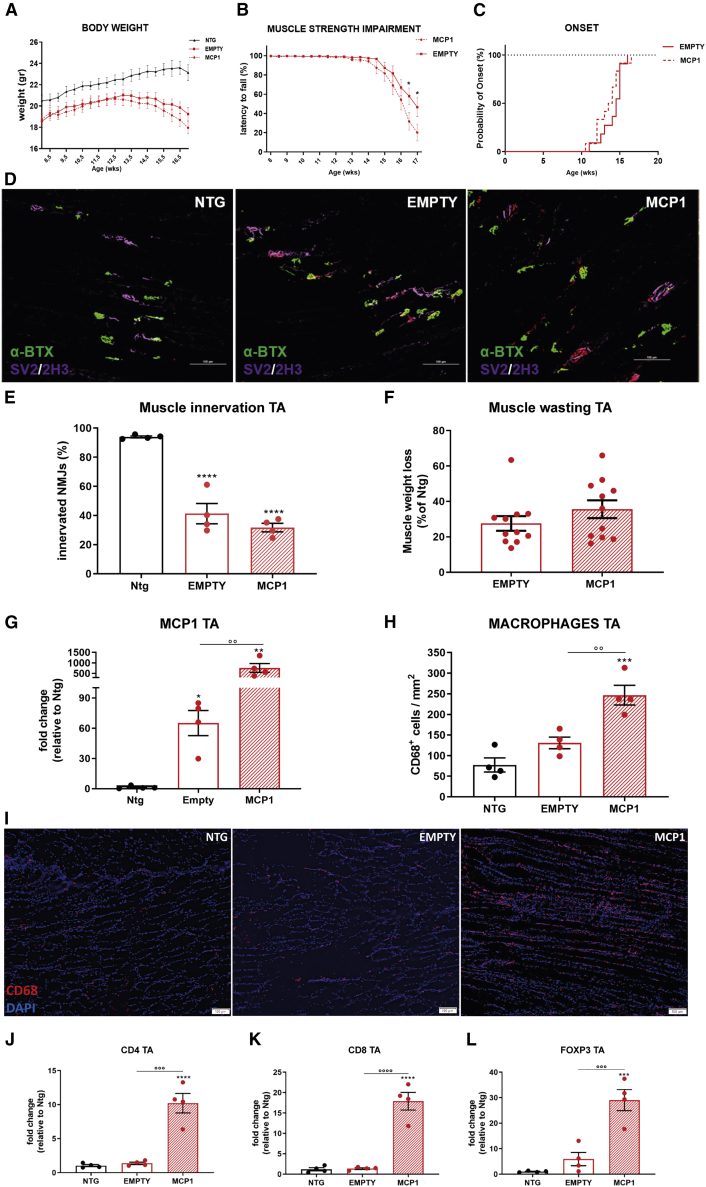
The scAAV9_MCP1 injection exacerbates the peripheral inflammatory response worsening the clinical phenotype of 129SvSOD1^G93A^ mice (A–C) Recording of (A) body weight and (B) muscle strength impairment of scAAV9_MCP1- and scAAV9(empty)-treated mice. Data are reported as means ± SEMs for each time point. ∗p < 0.05 by repeated-measures ANOVA with Sidak’s post-analysis. (C) No difference in disease onset was recorded between scAAV9_MCP1- and scAAV9(empty)-treated mice. p = 0.8899 by Mantel-Cox log rank test; n = 12 per group. (D) Representative micrographs of longitudinal TA muscle sections of scAAV9_MCP1- and scAAV9(empty)-treated mice and Ntg littermates at 17 weeks. α-Bungarotoxin (α-BTX, green): postsynaptic terminal; synaptic vesicle glycoprotein2A (SV2, purple) + neurofilament (2H3, purple): presynaptic bouton. Scale bar, 100 μm. (E) Neuromuscular junction count. Data are reported as means ± SEMs of 3–5 serial sections per muscle (approximately70 α-BTX^+^ endplates randomly taken) from n = 4 per group. (F) Muscle wasting was calculated by measuring the TA muscle weight of scAAV9_MCP1- and scAAV9(empty)-treated mice compared to relative Ntg littermates at 17 weeks. The percentage of muscle atrophy was calculated relative to Ntg mice. Data are reported as means ± SEMs; n = 12 per group. (G) Real-time PCR analysis of *Mcp1* transcript in the TA muscle of scAAV9_MCP1- and scAAV9(empty)-treated mice and Ntg littermates. Data are normalized to β-actin and expressed as means ± SEMs; n = 4 per experimental group. (H and I) Representative micrograph and relative quantification of TA muscle longitudinal sections of scAAV9_MCP1- and scAAV9(empty)-treated mice and Ntg littermates stained with the phagocytic marker CD68 (red) and DAPI (nucleus, blue). Scale bar, 100 μm. Data are reported as means ± SEMs of 3–5 serial sections per muscle from n = 4 per group. (J–L) Real-time PCR analysis of (J) *CD4*, (K) *CD8a*, and (L) *Foxp3* transcripts in the TA muscle of scAAV9_MCP1- and scAAV9(empty)-treated mice than Ntg littermates at 17 weeks. Data are normalized to β-actin and expressed as means ± SEMs; n = 4 per experimental group. ∗p < 0.05, ∗∗p < 0.01, ∗∗∗p < 0.001, ∗∗∗∗p < 0.0001 Ntg versus empty or MCP1; °°p < 0.01, °°°p < 0.001, °°°°p < 0.0001 empty versus MCP1 by 1-way ANOVA with Fisher post-analysis.

Eight-week-old 129SvSOD1^G93A^ mice (12 per group) were i.m. injected with the scAAV9_MCP1 and monitored until the clear symptomatic disease stage (17 weeks). The behavioral analysis showed that neither the body weight nor the motor onset (empty, 14.1 ± 0.4 weeks; MCP1, 13.4 ± 0.4 weeks [means ± SEMs]) was modified by the chemokine boosting (Figures 8A and 8C). Nevertheless, a worsening of the grip strength impairment was recorded in the scAAV9_MCP1-treated mice at the advanced disease stages (Figure 8B). However, the histological examination did not reveal any difference between the 2 groups of 129SvSOD1^G93A^ mice in the extent of NMJ denervation and TA muscle atrophy (Figures 8D–8F).

Compared to C57SOD1^G93A^ mice, at the symptomatic disease stage, fast-progressing mSOD1 mice strongly upregulated the chemokine within the TA muscle compared to respective Ntg littermates (mRNA fold change [FC] = 65.1 ± 12.4, 129SvSOD1^G93A^ mice; FC = 3 ± 0.2, C57SOD1^G93A^ mice [means ± SEMs]), which dramatically increased upon the scAAV9_MCP1 injection (FC = 752.1 ± 208.4 [mean ± SEM]) (Figure 8G).

Interestingly, the analysis of CD68^+^ cell density and *CD8a* and *CD4* transcripts demonstrated massive recruitment of macrophages and T lymphocytes in the hind paw muscle of the scAAV9_MCP1- but not the scAAV9(empty)-treated group compared with the Ntg littermates (Figures 8H–8K), suggesting a drastic alteration in the muscular inflammatory response in 129SvSOD1^G93A^ mice upon chemokine boosting. Notwithstanding the massive Foxp3^+^ Tregs infiltration (Figure 8L), an intense inflammation characterized the TA muscle of scAAV9_MCP1-treated mice, as demonstrated by the significant upregulation of the pro-inflammatory factors *Tnf-α* and gp91^PHOX^ compared with the control group (Figures S10A, S10C, and S10D). In addition, the unchanged Arg1 expression and the *Igf1* downregulation suggested that the infiltrated scAAV9_MCP1-recruited M1 macrophages were not apt to switch toward the M2 pro-healing phenotype^78^ (Figures S10A, S10B, and S10E). Notably, the MCP1 boosting and the eventual immune cell recruitment were unable to upregulate further the Pax7 and MyoG expression in 129SvSOD1^G93A^ mice (Figures S10A, S10F, and S10G).

Since in C57SOD1^G93A^ scAAV9_MCP1-treated mice the chemokine-mediated protective role arose early in the disease course, we assessed the effect of MCP1 boosting in fast-progressing mSOD1 mice approximately 2 weeks before the appearance of motor symptoms. Six-week-old 129SvSOD1^G93A^ mice (5 per group) were i.m. injected with the scAAV9 vectors, and the analysis of TA muscle was performed at 12 weeks of age. Intriguingly, although *Mcp1* resulted markedly upregulated (Figure S11C), 129SvSOD1^G93A^ mice appeared insensitive to chemokine boosting, as demonstrated by the unchanged CD68^+^ macrophage recruitment compared with the scAAV9(empty) group (Figures S11A and S11B). Indeed, no difference in the extent of the TA muscle atrophy was recorded between the 2 groups of 129SvSOD1^G93A^ mice at 12 weeks (Figure S11D).

Altogether, the data collected indicate a laggard activation of the muscular immune response by fast-progressing SOD1^G93A^ mice, culminating in an exacerbated inflammation upon MCP1 boosting that may be responsible for the worsened clinical phenotype at the advanced disease stage.

## Discussion

In this study, we examined the involvement of the MCP1-mediated axis in governing the speed of ALS progression in two SOD1^G93A^ models characterized by remarkable differences in the disease progression rate.

Our observations revealed that, albeit the scAAV9_MCP1 i.m. injection boosted the chemokine to the same extent along the neuromuscular system of the 2 ALS models, the treatment led to an opposite effect on the clinical phenotype of C57 compared with 129Sv mSOD1 mice. Slow-progressing C57SOD1^G93A^ mice responded positively to MCP1 boosting, anticipating the recruitment and phenotypic switch in leukocytes within the peripheral compartment. This sustained the activation of the myogenic program and nerve regeneration, finally slackening off the motor symptoms. Conversely, fast-progressing 129SvSOD1^G93A^ mice exhibited an adverse response to the treatment, exacerbating the toxic inflammatory response in the periphery, resulting in worsened motor ability late in the disease.

Intriguingly, our data showed a novel immune-unrelated role of MCP1 in promoting motor axon regeneration and modulating neuroinflammation in the nervous system of mSOD1 mice, with the overall effect of slackening MN degeneration.

We recently reported a different activation of MCP1 within MN soma and peripheral compartment of fast- versus slow-progressing SOD1^G93A^ models.^26^^,^^28^ Our studies revealed that fast-progressing mSOD1 mice exhibited earlier muscle denervation and motor axon deterioration correlated with lower immune cell infiltration in the peripheral compartment than slow-progressing ALS mice.^26^^,^^27^ We speculated that this defective immune response underpinned the greater peripheral degeneration and more rapid disease course of 129SvSOD1^G93A^ mice. This evidence put the MCP1-mediated immune cell recruitment forward as a discriminating factor of the different speed in the disease progression of the 2 SOD1^G93A^ models.

MCP1 is a chemokine with a renowned pro-inflammatory capability.^103^ In the neurological context, the increased expression of MCP1 is usually associated with neurodegenerative/neuroinflammatory diseases,^104^, ^105^, ^106^ including ALS.^107^, ^108^, ^109^ Accordingly, in the spinal cord of SOD1^G93A^ mice, we recorded a gradual increase in MCP1 levels as the disease progresses, characterized by a prominent expression by activated microglia at the advanced disease stage.

In addition to its classic toxic inflammatory activity, evidence indicated a pivotal role for the MCP1-mediated axis at orchestrating nerve^110^, ^111^, ^112^, ^113^ and muscle^29^^,^^49^^,^^68^^,^^114^ regeneration. In keeping with this, we recorded a gradual increase in chemokine expression along motor axons and Schwann cells as the disease progresses, suggesting the protective role of MCP1 in the PNS of mSOD1 mice.

Immune cell infiltration has been reported within nerves and skeletal muscles in ALS,^16^^,^^54^^,^^55^^,^^115^^,^^116^ although its contribution to the disease progression is still elusive. Here, we assessed the influence of immune response in the skeletal muscle of fast- and slow-progressing SOD1^G93A^ mice upon the i.m. injection of scAAV9_MCP1, which neatly boosted the chemokine along the motor unit of both ALS models.

Recent findings indicate that inflammatory response is coupled temporally and spatially to myogenesis, fulfilling a central role in bridging muscle initial injury responses and healing.^53^^,^^71^^,^^81^^,^^83^^,^^117^^,^^118^ The classic kinetics of the immune response in the skeletal muscle indicates that the first immune cells entering the damaged site possess a pro-inflammatory fingerprint, and they must switch toward the anti-inflammatory phenotype to accomplish tissue regeneration.^53^ In particular, while the pro-inflammatory response (“first wave”) is essential to phagocyte debris and stimulate SC proliferation, the transition toward the anti-inflammatory state (“second wave”) supports the formation and growth of new myofibers.^50^^,^^118^^,^^119^

As previously reported by Kunis et al.,^120^ mSOD1 mice exhibit a general immune deficiency, which can be rescued by boosting the immune response to promote the accumulation of inflammation-resolving cells in the CNS, finally ameliorating the disease progression. Accordingly, our data highlighted a deficient activation of the immune response also in the skeletal muscle of mSOD1 mice. At 14 weeks (i.e., approximately 2 weeks before overt muscle strength impairment), in concomitance with a pronounced TA muscle denervation atrophy, the C57SOD1^G93A^ scAAV9(empty)-treated mice are in the midst of the first (pro-inflammatory) wave of the immune response, in which neutrophils, the first immune cells entering within the damage site, amplify the inflammation and promote the recruitment of hematogenous leukocytes.^75^^,^^76^ Conversely, the scAAV9_MCP1 injection triggered the immune muscle response early in the disease leading to the second (pro-healing) wave at 14 weeks, in which pro-inflammatory cells (neutrophils, M1-macrophages, T cells) have given way to M2-macrophages and immunoregulatory T lymphocytes.^53^^,^^71^^,^^72^ In keeping with the *Tnf-α* and *Igf1* downregulation and increased Sirt1 expression,^78^^,^^79^ a higher percentage of CD206^+^ M2-macrophages was recorded in the hind paw muscle of scAAV9_MCP1-treated mice compared with the scAAV9(empty) group. In concerted action, the immunosuppressive capability of MCP1-recruited Tregs^121^ dampened the inflammation within the damaged tissue, sustaining the phenotypic switch of macrophages and the pro-healing mechanism.^74^^,^^122^^,^^123^ Ultimately, the lack of the inhibitory activity of neutrophils^124^ and inductive action of Tregs and M2-macrophages on myogenic progenitor cells^71^^,^^73^^,^^81^^,^^125^ sustained the tissue regeneration preserving the TA muscle from denervation atrophy and metabolic dysregulation. In addition, although at 20 weeks a reduced NMJs denervation was still recorded upon MCP1 boosting, the lack of the myogenic response in association with the same extent of TA muscle atrophy compared to C57SOD1^G93A^ scAAV9(empty)-treated mice indicated a gradual exhaustion of the elicited muscle pro-healing immune response in the hindlimbs of ALS mice as the disease progresses. Nevertheless, the preservation of the forepaw muscles, which are belatedly affected in the mSOD1 model,^62^^,^^100^^,^^101^ may be partially responsible for the ameliorated motor performance of C57SOD1^G93A^ scAAV9_MCP1-treated mice at the advanced disease stage.^102^ The early MCP1 boosting within TB muscle of slow-progressing mSOD1 mice forced and sustained the activation of the pro-inflammatory response within a less harmed tissue, promoting the recruitment of macrophages and cytotoxic T lymphocytes at 14 weeks. The early leukocyte recall was decisive at countenancing the transition of the immune muscle response toward the anti-inflammatory and pro-regenerative state^50^^,^^119^ at the symptomatic disease stage, eventually preventing the forepaw muscle denervation atrophy.

The evidence herein collected highlighted the pivotal role of the peripheral immune response in triggering skeletal muscle regeneration and its temporal activation as a limiting factor in achieving a significant effect to slacken off the disease progression in mSOD1 mice.

In support of this evidence, fast-progressing SOD1^G93A^ mice, whose genetic background is associated with a poor ability to recruit immune cells during phlogosis,^126^^,^^127^ showed a delayed activation of the pro-inflammatory immune muscle response upon MCP1 boosting that exacerbated the disease severity. Indeed, although the chemokine was massively upregulated 6 weeks after the scAAV9_MCP1 injection, 129SvSOD1^G93A^ mice were unable to promptly and properly react to the chemotactic gradient established within the TA muscle fostering the hematogenous macrophage recruitment. The defective activation of the immune response early in the disease led to its mismanagement at the advanced stage, hindering the correct immune response kinetics. Pursuant to the dramatic macrophages and T lymphocytes recruitment, at 17 weeks, persistent inflammation characterized the TA muscle of 129SvSOD1^G93A^ scAAV9_MCP1-treated mice despite the massive Tregs recall, arguably due to their impaired immunomodulatory capability at the advanced disease stage.^97^ Finally, the tardive leukocyte recruitment inhibited the immune muscle response switch toward the anti-inflammatory fingerprint, eventually hampering skeletal muscle regeneration and function.^53^^,^^119^ This result mirrored the recent findings by Rizzo et al.,^128^ who demonstrated that in splenectomized *mdx* mice, the delayed macrophage infiltration impaired their shift toward the M2 pro-healing fingerprint, eventually hindering muscle fiber regeneration. Therefore, we can surmise that the tardive and maladaptive activation of the peripheral immune response, even upon MCP1 boosting, may be the chief culprit of the faster disease progression of 129SvSOD1^G93A^ mice.

The data collected in the hind paw muscle of C57SOD1^G93A^ mice suggested that preserving the muscular compartment since the early disease stage may have slackened off ALS dying-back degeneration of the motor system.^11^ Nevertheless, we demonstrated that the retrograde overexpression of MCP1 within the sciatic nerves of mSOD1 mice directly affected the stability and regeneration of peripheral motor axons. Indeed, the chemokine upregulation within the sciatic nerve of C57SOD1^G93A^ scAAV9_MCP1-treated mice resulted in increased sprouting of GAP43^+^ motor axon terminal branches, accounting for the reduced NMJ denervation of hindlimb muscles across the disease progression.

The dispensable capability of MCP1 to promote axonal outgrowth was previously described in axonal Survival Motor Neuron (aSMN)-expressing NSC34 cell cultures^44^ and dorsal root ganglia (DRG) explants obtained from MCP1-treated^112^^,^^129^ or genetically depleted mice.^111^ According to this information, the chemokine plays an immune-unrelated role in amplifying and maintaining the regenerative capacity of peripheral axons, promoting the expression of the regeneration-associated genes (e.g., GAP43), which is concurrent to the MCP1-mediated neuron-macrophage interaction.^45^^,^^111^^,^^129^

Based on our evidence, the MCP1 pro-regenerative effect in the PNS of slow-progressing mSOD1 mice became clearer at the symptomatic disease stage, when the chemokine overexpression preserved the Schwann cell-axon unit, decreased toxic inflammation, and sustained motor axon sprouting, inferring an action directly mediated by the transduced MNs rather than a straight chemokine activity on the Schwann cells.

Although the MCP1 pleiotropic mechanism is far from being elucidated, recent studies reported the direct influence of the chemokine at modulating the neuroinflammation by governing the fingerprint of the recruited myeloid cells. For instance, several observations obtained in animal models of spinal cord injury demonstrated that MCP1 released by neurons attracts and activates macrophages through CCR2 to drive them toward the M2 pro-healing phenotype. In turn, MCP1-activated macrophages establish a permissive environment,^111^^,^^129^ eventually preventing neurodegeneration.^45^ Here, we showed that a similar mechanism could be prompted within the spinal cord of SOD1^G93A^ mice, where MCP1 was significantly upregulated by MNs at the onset of the disease. Although it is now clear that hematogenous monocytes cannot penetrate the CNS of mSOD1 mice,^16^^,^^120^^,^^130^ we can suppose that the scAAV9-specific induction of MCP1 within the MN perikaryon may have modulated the activation state of the CNS-resident myeloid cells (i.e., microglia). Suitably, our data demonstrated that the scAAV9_MCP1 injection in pre-symptomatic mSOD1 mice extended the “stable phase” of the disease,^96^^,^^97^ maintaining the M2 polarization of the neuroinflammatory milieu and reducing MN loss along the disease course.

Altogether, the data collected suggested that the protective effect recorded in the slow-progressing C57SOD1^G93A^ scAAV9_MCP1-treated mice resulted from the dual chemokine function within the motor unit. On the one hand, the early MCP1 boosting promoted and sustained the immune muscle response and, consequently, slackened the dying-back degeneration. On the other hand, the immune unrelated MCP1 capability within the neuron perikaryon and motor axons modulated the neuroinflammatory phenomenon and promoted axonal regeneration preserving MN, finally slowing down the dying-forward degeneration.

### Conclusions

For the first time in the ALS context, we demonstrated the pivotal role of the immune response in promoting and governing skeletal muscle regeneration and thus the speed of the disease progression. Our observations suggest that, although potentially protective, the immune response is delayed in ALS mice and, hence, is ineffective at sustaining a substantial recovery of the peripheral compartment. Notably, the dichotomic effect recorded in the 2 SOD1^G93A^ strains following MCP1 boosting pointed out the nature and temporal activation of the immune response as discriminating factors to foster skeletal muscle regeneration, slackening the dying-back degeneration and slowing down ALS course. This also emphasizes the different immune response due to genetic background as a key determinant of the variability in the disease progression as reported in ALS patients carrying the same SOD1 mutation.^3^^,^^4^ Altogether, these observations nominate the muscular compartment as a primary target for developing effective therapeutic interventions in ALS capable of interfering with the speed of the symptoms progression and the dying-back degeneration tangibly. In addition, the comprehension of the mechanisms underlying the protective role fulfilled by MCP1 in the motor unit of mSOD1 mice may provide innovative evidence regarding the contribution of the immune response in ALS.

Despite *in vitro* and *in vivo* models of the disease having generated different potential pharmacological targets, ALS still lacks an adequate therapy able to delay or even halt its development.^131^^,^^132^ We think this is due mainly to the poor knowledge of the temporal and spatial mechanisms by which the immune response governs the pattern of the disease.^133^^,^^134^ Our findings provide a possible explanation for the failure of untargeted immunomodulatory treatments^23^^,^^135^ and suggest that the characterization of the immune muscle profile in patients may be a clinical adjunct to improve clinical practice and develop innovative and personalized strategies to hinder ALS progression.

Altogether, the evidence provided herein demonstrated the crucial role of the thus-far underestimated peripheral compartment in ALS pathoprogression straightforwardly. Although the latest clinical studies have reported a defective monocyte/macrophage infiltration at the site of nerve degeneration^136^ and a direct correlation between the PNS inflammation and longer disease duration,^21^ no observations are available on a massively affected body compartment in ALS: the skeletal muscle. Therefore, in view of the easy accessibility of bioptic samples, the characterization of the immune muscle fingerprint to assess a potential correlation with biomolecular pathways underlying atrophy and myogenesis may produce a combination of muscle-derived, immune-related molecular signatures that will be useful as a clinical adjunct in the prognostic evaluation of ALS patients.

## Materials and methods

### Mice

Female transgenic SOD1^G93A^ mice on C57BL/6J (stock no. 004435; The Jackson Laboratories, Bar Harbor, ME, USA) or 129SvHsd genetic background, hereafter indicated as C57SOD1^G93A^ and 129SvSOD1^G93A^, respectively, and corresponding Ntg littermates were used. Transgenic SOD1^G93A^ mice expressing approximately 20 copies of mutant human SOD1 with a Gly93Ala substitution (B6SJL-TgSOD1^G93A^-1Gur) were initially obtained from The Jackson Laboratories and maintained on a C57BL/6JOlaHsd (C57) genetic background at Harlan Italy S.R.L. (Bresso, Milan, Italy). From the crossbreeding of C57BL/6JOlaHsd (C57SOD1^G93A^) with 129S2/SvHsd (129Sv) for >15 generations, we obtained SOD1^G93A^ mice on the homogeneous background 129SSv (129SvSOD1^G93A^).

All of the animal procedures have been performed according to the following laws, regulations, and policies governing the care and use of laboratory animals: Italian Governing Law (D.lgs 26/2014; Authorization no. 19/2008-A, issued March 6, 2008 by the Ministry of Health); Mario Negri Institutional Regulations and Policies providing internal authorization for persons conducting animal experiments (Quality Management System Certificate, UNI EN ISO 9001:2015 – regulation no. 6121); the National Institutes of Health’s (NIH) *Guide for the Care and Use of Laboratory Animals* (2011 edition); and EU directives and guidelines (EEC Council Directive 2010/63/UE). Four to five mice were housed per standard cage in specific pathogen-free and controlled environmental conditions (temperature: 22°C ± 2°C; relative humidity: 55% ± 10%; and 12 h of light). Food (standard pellets) and water were supplied *ad libitum*.

### Intramuscular administration of scAAV9 vector

Engineered (gfp or mcp1) or empty scAAV9 vectors were purchased by Virovek (Hayward, CA, USA). To ensure a high expression of the transgene (gfp or mcp1), the constitutive CMV promoter was used.

Adult (6 or 8 weeks old) SOD1^G93A^ mice underwent a single bilateral i.m. injection of 2.18 × 10^10^ vg/μL of the scAAV9 vector. The scAAV9, opportunely diluted in sterile PBS, was injected in both hindlimb (TA, GCM, and GM) and forelimb (TB) muscles following the protocol previously described by Gruntman et al.^137^ Briefly, mice were anesthetized with isoflurane inhalation, fur shaved to visualize the target muscles, and a 30-G needle was inserted in the muscle center to inject the scAAV9 (10 μL/muscle). Mice were divided into the treated (scAAV9_MCP1) and control (scAAV9_(empty)) groups through a block randomization in which the blocks are defined by the body weight, sex, and sibling separation.

### Behavioral analysis

Starting from 8 weeks of age, motor onset and disease progression were monitored biweekly in C57SOD1^G93A^ and 129SvSOD1^G93A^ scAAV9-treated mice by a blinded operator recording the body weight and the motor performance at the paw grip endurance (PaGE) test. In the PaGE test, mice are placed on a horizontal grid at a 30-cm height, and the tail is gently pulled until the mice grasp the grid with their fore and hind paws. The grid is gently turned upside down, and the latency time of the mouse to fall on the table is recorded for a maximum of 90 s. Each mouse is given up to three attempts, and the most prolonged latency is recorded. The onset of muscle strength deficit is considered when the mice showed the first signs of impairment in the PaGE test. In C57SOD1^G93A^ mice, the latency was evaluated as previously described;^102^ conversely, in 129SvSOD1^G93A^ animals, the performance obtained in the grip strength test was assessed through a score, calculated as indicated by Lauranzano et al.^138^

### Immunohistochemical (IHC) analysis

Mice were anesthetized with a mix of ketamine (1.75 mg/kg) and medetomidine (1 mg/kg) and transcardially perfused with 50 mL 0.1 M PBS, pH 7.4. Following blood removal, the skeletal muscles (TA, GCM, GM, and TB) and nerves were dissected and immediately frozen in cooled isopentane or n-pentane, respectively. At the same time, the vertebral column was post-fixed overnight in a solution of 4% paraformaldehyde in 0.1 M PBS. The following day, the vertebrae were removed, and the spinal cord was transferred to 30% sucrose solution with 0.1% sodium azide in 0.1 M PBS at 4°C for cryoprotection before mounting in optimal cutting temperature compound (Tissue-Tek, Sakura Finetek, Torrance, CA, USA).

The following primary antibodies and staining were used: chicken anti-GFP (1:750; GTX13970, GeneTex, Irvine, CA, USA); rat anti-MCP1 (1:50; ab8101, Abcam [Cambridge, UK]); rabbit anti-Iba1 (1:500; 019–19741, Fujifilm Wako, Osaka, Japan); mouse anti-GFAP (1:2,500; MAB3402, Merck Millipore, Burlington, MA, USA); goat anti-ChAT (1:200; AB144P Merck Millipore); rat anti-macrosialin (CD68, 1:200; MCA1957, BioRad, Hercules, CA, USA); Neurotrace conjugated with Alexa Fluor 647 (1:500; N21483, Invitrogen, Waltham, MA, USA); mouse anti-S100β (1:400; AMAB91038, Sigma-Aldrich, St. Louis, MO, USA); mouse anti-phosphorylated neurofilament H (Smi31, 1:5,000; 801608, BioLegend, San Diego, CA, USA); rabbit anti-neurofilament heavy polypeptide (NF200, 1:1,000; N4142, Sigma-Aldrich); rat anti-CD11b (1:200; MCA74G, BioRad); rabbit anti-iNOS (1:200; PA3-030A, Invitrogen); rabbit anti-mannose receptor (CD206, 1:200; ab64693, Abcam); mouse anti-Pax7 (1:400; AB_528428, Developmental Studies Hybridoma Bank [DSHB], Iowa City, IA, USA); rabbit anti-MyoD (1:100; PA5-23078, Invitrogen); rabbit anti-neutrophil elastase (1:300; ab68672, Abcam); Hoechst (1:1,000; Roche, Basel, Switzerland). Alexa Fluor 488, 594, and 647 secondary antibodies (Invitrogen) were used at a dilution of 1:500. All of the immunohistochemistry was done following an indirect immunostaining protocol at room temperature, except for primary antibody staining, which was performed at 4°C overnight.

Spinal cord immunohistochemistry was done on free-floating sections (30 μm), and then mounted on glass slides (Waldemar Knittle) with Fluorsave (Calbiochem). Cryosections of nerves (14 μm) or skeletal muscles (20 μm, longitudinal; 12 μm, coronal) were treated directly on polylysine objective slides (VWR International, Radnor, PA, USA) and then mounted with Fluorsave (Calbiochem, San Diego, CA, USA).

Fluorescence-labeled spinal cord sections were analyzed under a sequential scanning mode to avoid bleed-through effects with an IX81 microscope equipped with a confocal scan unit FV500 with three laser lines: Ar-Kr (488 nm), He-Ne red (646 nm), and He-Ne green (532 nm) (Olympus, Tokyo, Japan) and a UV diode using a 10× objective. For lumbar motor neurons count (1 every 10 sections), a total of 12 serial choline acetyltransferase (ChAT)-stained sections were analyzed. The neuron areas were analyzed with Fiji software (ImageJ, NIH, Bethesda, MD, USA). As previously indicated,^139^ only neuronal somas with an area ≥400 μm^2^ were considered for the quantitative analysis of MN numbers. Fluorescence-labeled sections images (3–5 per animal) of the TA and TB muscle were analyzed with an Olympus virtual slide system VS110 (Olympus, Center Valley, PA, USA) and acquired at 20× magnification. A systematic random sampling procedure was applied as previously described.^102^^,^^140^ Briefly, a grid of equivalent sampling fields was outlined on the muscle slice profile. To ensure that every part of the slice had an equal chance of being sampled, a bidimensional stereological sampling procedure was applied analyzing equivalent fields placed at a fixed distance from each other on the tissue slice, using the “grid” function in Fiji (ImageJ). The same approach was used to evaluate the neutrophil elastase staining by calculating the percentage of covered area (area fraction percentage) per field for each section in the analysis with Fiji software.

### Morphometric analysis of muscles

TA and TB muscles were dissected out and snap-frozen in liquid nitrogen. For the muscle fiber composition (SDH staining), 10 μm-thickness serial coronal cryosections from the mid-belly region of the TA muscle were air-dried and then incubated at 37°C for 30 min in phosphate buffer (0.2 M, pH 7.6) containing 13.5 mg/mL Na-succinate (Sigma-Aldrich) and 0.5 mg/mL nitro blue tetrazolium (Sigma-Aldrich, 0.29 mg/mL buffer solution). After staining, sections were fixed with 4% paraformaldehyde, dehydrated in 15% alcohol for 5 min, and finally mounted with DPX compound (Sigma-Aldrich).

For the muscle fiber cross-sectional area, 10-μm thickness serial coronal cryosections from the mid-belly region of the TA muscle were air-dried, fixed in 4% paraformaldehyde solution for 5 min, and stained with wheat germ agglutinin, Alexa Fluor 488 conjugate (1:500; W11261, Thermo Fisher, Pittsburgh, PA, USA) and Hoechst (1:1,000; Roche).

Images were acquired with an Olympus virtual slide system VS110 (Olympus, Center Valley, USA) at 20× magnification and analyzed through Fiji (ImageJ) on 3–5 serial sections per animal. For the SDH staining, a systematic random sampling procedure was applied as described above. For the muscle fiber cross-sectional area and centralized nuclei, the entire TA or TB muscle section was analyzed with the MuscleJ plug-in of Fiji software, as previously described.^141^

### Muscle denervation and terminal motor axons sprouting

TA muscles were dissected out and snap-frozen in liquid nitrogen. Six serial longitudinal cryosections (20-μm-thickness) per animal were analyzed. Muscle sections were stained with mouse antisynaptic vesicle glycoprotein 2A (SV2, 1:50; SV2, DSHB), mouse anti-neurofilament medium polypeptide (2H3, 1:100; AB_2314897, DSHB) and rabbit anti-GAP43 (1:100;^142^) following a classic indirect staining protocol. α-Bungarotoxin (α-BTX) coupled to Alexa Fluor 594 (1:500; B13423, Invitrogen) was incubated for 1 h at room temperature. Images were obtained with Nikon A1 confocal scan unit (Nikon, Tokyo, Japan) at 20× magnification. The co-localization channel between neurofilament (SV2/2H3), GAP43 and α-BTX immunostaining was produced for each *Z* stack. The percentage of innervated NMJs was quantified considering the overlap between neurofilament (SV2/2H3) staining and α-BTX labeled endplates. The regenerating terminal motor axon rate was calculated based on the co-localization between neurofilaments (SV2/2H3), GAP43 staining, and α-BTX labeled endplates. The analyses were performed by NIS elements software (Nikon, Tokyo, Japan).

### Western blot

Mice were anesthetized with a mix of ketamine (1.75 mg/kg) and medetomidine (1 mg/kg) and transcardially perfused with 50 mL 0.1 M PBS, pH 7.4. Following blood removal, skeletal muscles were dissected out and immediately frozen in cooled isopentane. The spinal cord was fluxed from the vertebral column using sterile physiological solution (0.9% NaCl) and dissected in the three main segments (i.e., cervical, thoracic, and lumbar). Spinal cord segments and nerves were immediately frozen on dry ice. Protein lysates were obtained by homogenizing the skeletal muscles, sciatic nerves, and spinal cords of mice in lysis buffer, as previously described.^102^ Briefly, tissues were powdered in liquid nitrogen, homogenized by sonication in ice-cold homogenization buffer (Tris HCl, pH 8, 50 mM, NaCl 150 mM, EGTA pH 8.5 mM, MgCl_2_ 1.5 mM, Triton X-100 1%, anhydrous glycerol 10%, phosphatases and proteases inhibitor cocktail; Roche), and centrifuged at 13,000 rpm for 15 min at 4°C, and the supernatants were collected and stored at −80°C.

Equal amounts of total protein homogenates were loaded on polyacrylamide gels and electroblotted onto polyvinylidene fluoride (PVDF) membrane (Millipore) using the Trans-Blot Turbo Transfer System (BioRad). After saturation with blocking agent, membranes were immunoblotted with the following primary antibodies: chicken anti-GFP (1:5,000, GTX13970 GeneTex); mouse anti-glyceraldehyde 3-phosphate dehydrogenase (GAPDH) (1:10,000; CB1001, Merck Millipore); mouse anti-β-actin (1:30,000; MAB1501, Merck Millipore); rabbit anti-arginase1 (1:1,000; ab91279, Abcam); mouse anti-gp91^PHOX^ (1:1,000; 611415, BD Biosciences, Franklin Lakes, NJ, USA); mouse anti-GFAP (1:30,000; MAB3402, Merck Millipore); rabbit anti-Iba1 (1:1,000; 019–19741, Fujifilm Wako); goat anti-p75^NTR^ (1:1,000; sc-271708, Santa Cruz Biotechnology, Dallas, TX, USA); rabbit anti-NF200 (1:4,000; N4142, Sigma-Aldrich); rat anti-MBP (1:1,000; aa82-87, BioRad), mouse anti-SIRT1 (1:750; S5196, Sigma-Aldrich), mouse anti-Pax7 (1:1,000; AB_528428, DSHB), rabbit anti-MyoD (1:5,000; PA5-23078, Invitrogen); mouse anti-MyoG (1:350; AB_2146602, DSHB) followed by horseradish peroxidase (HRP)-conjugated secondary antibodies (Thermo Fisher) and developed with Luminata Forte Western Chemiluminescent HRP Substrate (Millipore) at ChemiDoc Imaging Systems (BioRad). The optical density of the blots was measured with Image Lab 6.1 software (BioRad) and normalized to the total amount of protein loaded stained with Ponceau S solution (Sigma-Aldrich),^143^ unless otherwise specified.

### Real-time PCR

Tissues (spinal cords, sciatic nerves, and muscles) were freshly collected and immediately frozen on dry ice after mouse perfusion with 0.1 M PBS. The total RNA from tissues was extracted using the Trizol method (Invitrogen) and purified with PureLink RNA columns (Thermo Fisher) following the manufacturer’s instructions. RNA samples were treated with DNase I, and reverse transcription was done with a High-Capacity cDNA Reverse Transcription Kit (Thermo Fisher).

For real-time PCR, we used the TaqMan gene expression assay (Applied Biosystems, Waltham, MA, USA) following the manufacturer’s instructions on cDNA specimens in triplicate, using SensiFAST Probe Hi-ROX Kit (BioLine International, Toronto, Canada) and 1x mix containing the specific probes (Thermo Fisher). The following probes (Thermo Fisher) were used for the real-time PCR assay: MCP1 (Mcp1, Mm00441242_m1); cholinergic receptor nicotinic gamma subunit (Chrng, Mm00437419_m1); CD8 alpha receptor (CD8a; Mm01182107_g1); CD4 alpha receptor (CD4a; Mm00442754_m1); Forkhead box P3 (Foxp3; Mm00475162_m1); *Igf1* (Mm00439560_m1); Tnf-α (Mm00443258_m1); macrosialin (CD68; Mm03047343_m1); *Il4* (Mm00445259_m1); and Il1β (Mm00434228_m1). Relative quantification was calculated from the ratio between the cycle number (Ct [cycle threshold]) at which the signal crossed a threshold set within the logarithmic phase of the given gene and that of the reference β-actin gene (Mm02619580_g1). Mean values of the triplicate results for each animal were used as individual data for the Livak relative gene expression analysis (2^−ΔΔCt^).

### Statistical analysis

All of the statistical analyses were performed using Prism 9 for Windows (GraphPad Software, San Diego, CA, USA). Values are reported as means ± SEMs. For each analysis, the dependent and group variable are named on the y- and x axis of the graph, respectively.

The sample size for behavioral analysis was defined according to the guidelines for preclinical animal research in ALS/MN disease as reported by Ludolph et al.^144^ Parameters (body weight and PaGE test) used to evaluate disease progression in SOD1^G93A^ mice were analyzed by repeated-measures ANOVA followed by Sidak’s post-analysis. Symptoms onset was analyzed by log rank Mantel-Cox test, and Kaplan-Meier plots were generated.

Mean values ±standard deviations were used for statistical analysis by Student’s t test for two groups or by one-way ANOVA followed by Fisher’s multiple comparison test for more than two groups. In the case of two independent variables, two-way ANOVA followed by Fisher’s least significant difference (LSD) multiple comparison test was performed. The D’Agostino-Pearson omnibus normality test and relative QQ plots were used to assess the assumption of normality. In the event of populations with unequal variance, the Brown-Forsythe ANOVA test followed by the unpaired t test with Welch’s correction was applied.

For all of the analyses, a p value <0.05 was considered statistically significant. The asterisk indicates the comparison with the Ntg littermates, while the dot (°) indicates the comparison between scAAV9_MCP1- and scAAV9_(empty)-treated mice. Further details, including p values and number of samples, are documented in the Results, figures, and relevant captions.

## Supplemental information

Document S1. Figures S1–S11 and Table S1

Document S2. Article plus supplemental information
