# Supplementary material for: Boosting the peripheral immune response in the skeletal muscles improved motor function in ALS transgenic mice
Source: Mol Ther. 2022 Apr 27;30(8):2760–84. doi: 10.1016/j.ymthe.2022.04.018 (PMC9372324; doi:10.1016/j.ymthe.2022.04.018)
Supplement: Document S1. Figures S1–S11 and Table S1 [file mmc1.pdf]

YMTHE, Volume *30*

Supplemental Information

Boosting the peripheral immune response in the skeletal muscles improved motor function in ALS transgenic mice

Maria Chiara Trolese, Carlotta Scarpa, Valentina Melfi, Paola Fabbrizio, Francesca Sironi, Martina Rossi, Caterina Bendotti, and Giovanni Nardo

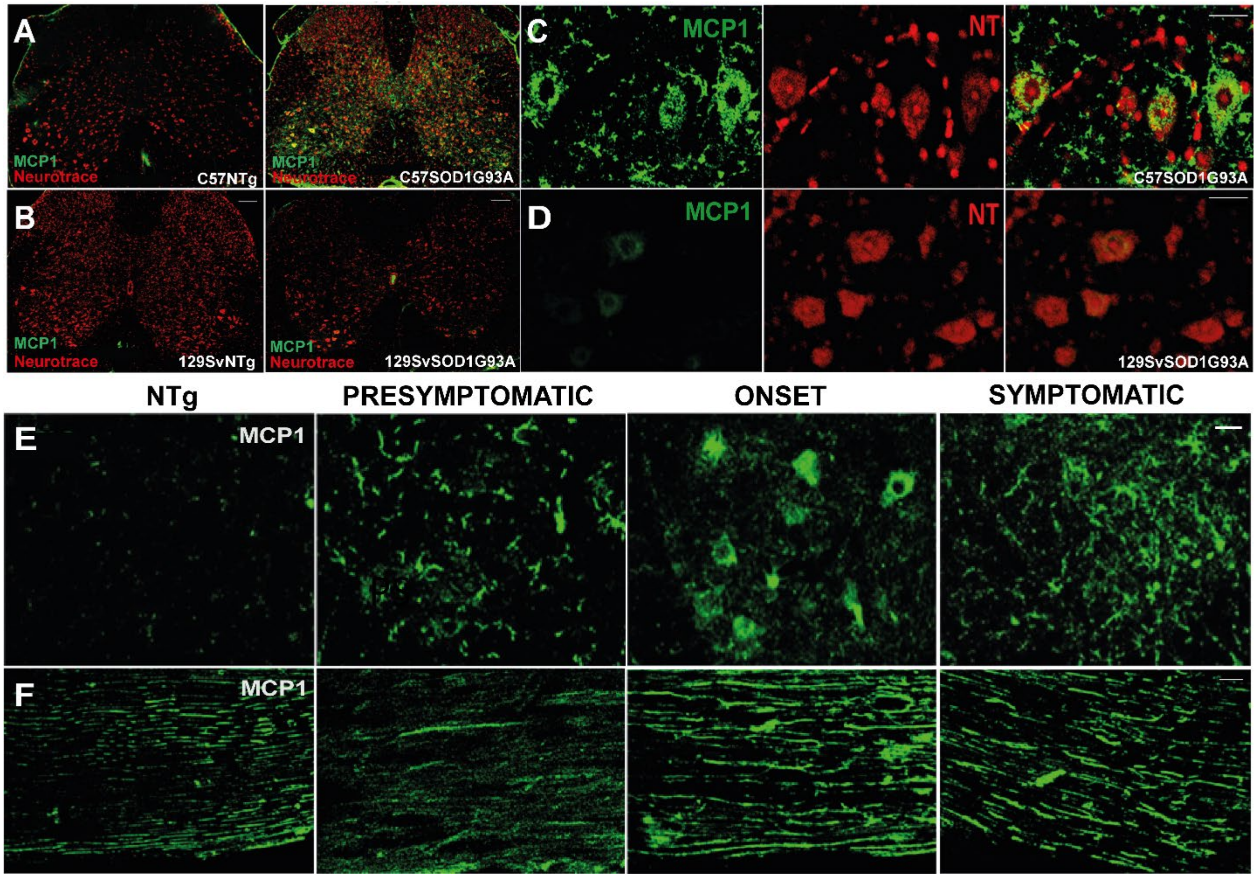

Figure S1_ ***MCP1 expression pattern in the CNS and PNS of fast- and slow-progressing SOD1^{G93A} mice.*** **A-D** Confocal micrograph of MCP1 stained (green) coronal section of the lumbar spinal cord of (A, C) C57SOD1^{G93A} and (B, D) 129SvSOD1^{G93A} mice at the disease onset (NT, neurotrace; red). Scale bar, (A, B) 100µm; (C, D) 50µm. **E, F** MCP1 expression gradually increases in the lumbar spinal cord (E) and sciatic nerve (F) of C57SOD1^{G93A} mice as the disease progresses. Scale bar, 100µm. The qualitative immunofluorescence analysis was performed on 4/6 serial sections of the L3-L5 spinal cord or sciatic nerve from at least n=3 mice per strain and genotype at each time point.

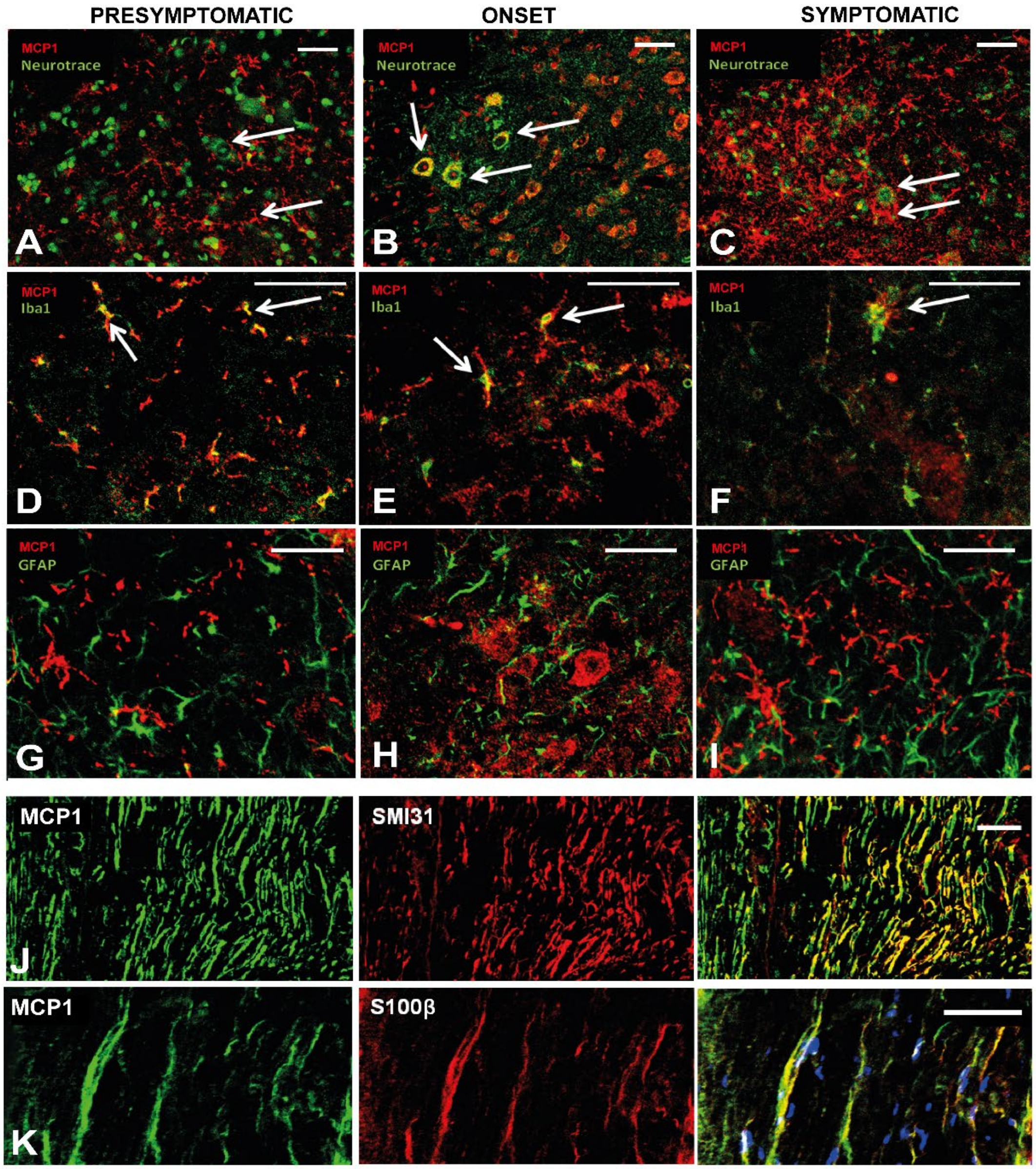

Figure S2_ ***MCP1 expression pattern in the CNS and PNS of C57SOD1^{G93A} mice during disease progression.*** **A-I** Confocal micrographs of coronal sections of the lumbar spinal cord of C57SOD1^{G93A} mice at the pre-symptomatic, onset and symptomatic disease stage stained with MCP1 (red) and (A-C) Neurotrace (MNs, green) or (D-F) Iba1 (microglia, green) or (G-I) GFAP (astrocytes, green). Scale bar, (A-C, G, H, I) 50μm; (D-F) 20μm. **J, K** Confocal micrographs of longitudinal sections of the sciatic nerve of C57SOD1^{G93A} mice at the disease onset stained with MCP1 (green) and (J) SMI31 (motor axons, red) or (K) S100β (Schwann cells, red). Nuclei are stained with DAPI (blue). Scale bar, (J) 50μm; (K) 20μm. The qualitative immunofluorescence analysis was performed on 4/5 serial sections of the L3-L5 spinal cord or sciatic nerve from at least n=3 mice at each time point.

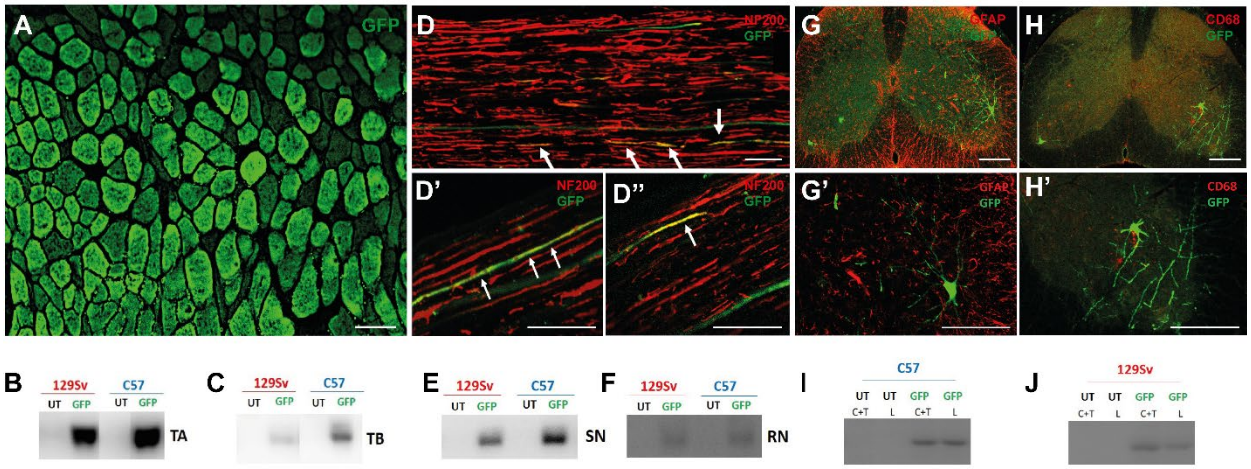

Figure S3_ *A single scAAV9_GFP i.m. injection mediates widespread motor unit transduction in adult SOD1^{G93A} mice.* **A-C** Analysis of GFP expression in skeletal muscle. (A) Confocal micrograph of TA muscle coronal section stained with Green Fluorescent Protein (GFP, green). Scale bar, 50µm. Representative immunoblot images of GFP expression in (B) TA and (C) TB muscle extracts of adult scAAV9_GFP-treated compared with untreated C57 and 129Sv SOD1^{G93A} mice. **D-F** Analysis of GFP expression in peripheral nerves. (D) Confocal micrographs of sciatic nerve longitudinal sections stained with GFP (green) and heavy neurofilament (NF200, red). The inset shows a motor axon expressing GFP (arrows). Scale bar, (D) 100µm; (D', D'') 20µm. Representative immunoblot images of GFP expression in the (E) sciatic and (F) radial nerve extracts of adult scAAV9_GFP-treated compared with untreated C57 and 129Sv SOD1^{G93A} mice. **G-J** Analysis of GFP expression in the lumbar spinal cord. (G-H') Confocal micrographs of coronal sections of the lumbar spinal cord stained with GFP (green), (G, G') GFAP (astrocytes, red) or (H, H') CD68 (microglia, red). (G', H') The insets show a specific GFP expression within MN perikarya but not by non-neuronal neighbouring cells. Scale bar, (G, H) 50µm; (G', H') 20µm. Representative immunoblot images of GFP expression in the lumbar and cervical segment of the spinal cord of adult (I) C57 and (J) 129Sv SOD1^{G93A} scAAV9_GFP-treated or untreated mice. (GFP, scAAV9_GFP-treated; UT, untreated; TA, *Tibialis Anterior*; TB, *Triceps Brachii;* SN, sciatic nerve; RN, radial nerve; C+T, cervical and thoracic spinal cord; L, lumbar spinal cord). The qualitative immunofluorescence analysis was performed on 4/6 serial sections of the TA muscle mid-belly region, sciatic nerve, or L3-L5 spinal cord from at least n=3 mice per strain and treatment group. The immunoblot analysis was performed on TA and TB muscle, sciatic and radial nerve, lumbar and cervical spinal cord extracts from at least n=3 mice per strain and treatment group.

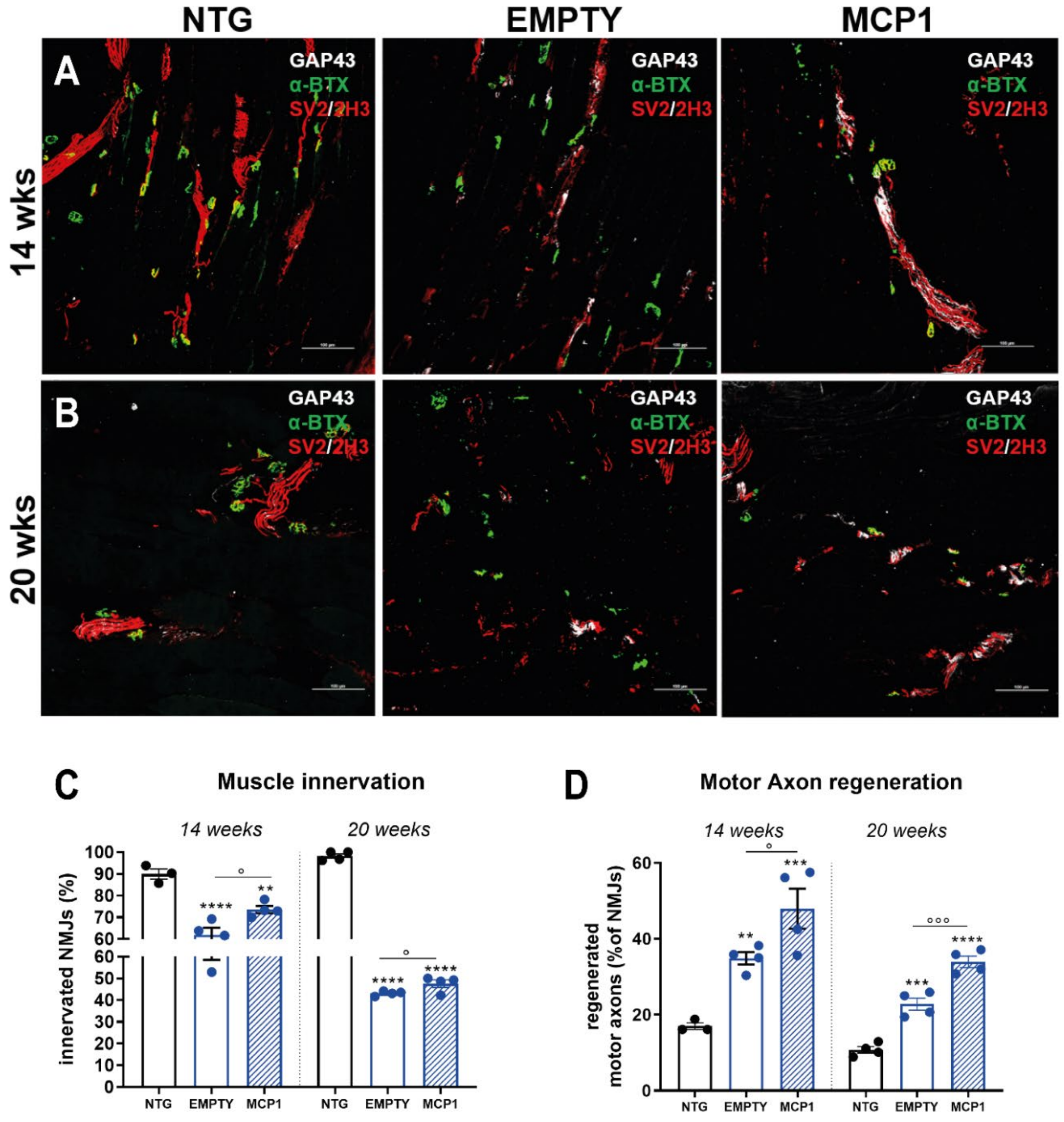

Figure S4_ *The MCP1-mediated immune response prevents muscle denervation and promotes axonal sprouting in C57SOD1^{G93A} mice.* **A, B** Representative micrographs of longitudinal TA muscle sections of scAAV9_MCP1- and scAAV9(empty)-treated mice and Ntg littermates at (A) 14 and (B) 20 weeks. α-Bungarotoxin (αBTX, green): postsynaptic terminal; synaptic vesicle glycoprotein2A (SV2, red) + neurofilament (2H3, red): presynaptic bouton; growth-associated protein 43 (GAP43, white): regenerating motor axons. Scale bar, 100μm. **C, D** The relative quantifications show the significant preservation of (C) the neuromuscular junction (NMJ) innervation and (D) a higher axonal regeneration in the TA muscle of scAAV9_MCP1-treated mice compared with the scAAV9(empty) group. Data are reported as mean±SEM of 3/5 serial sections per muscle (~70 α-BTX$^+$ endplates randomly taken) from n=4 mice per experimental group at each time point. **$p<0.01$, ***$p<0.001$, ****$p<0.0001$ Ntg Vs EMPTY or MCP1; °$p<0.05$, °°°$p<0.001$ EMPTY Vs MCP1 by one-way ANOVA with Fisher post-analysis.

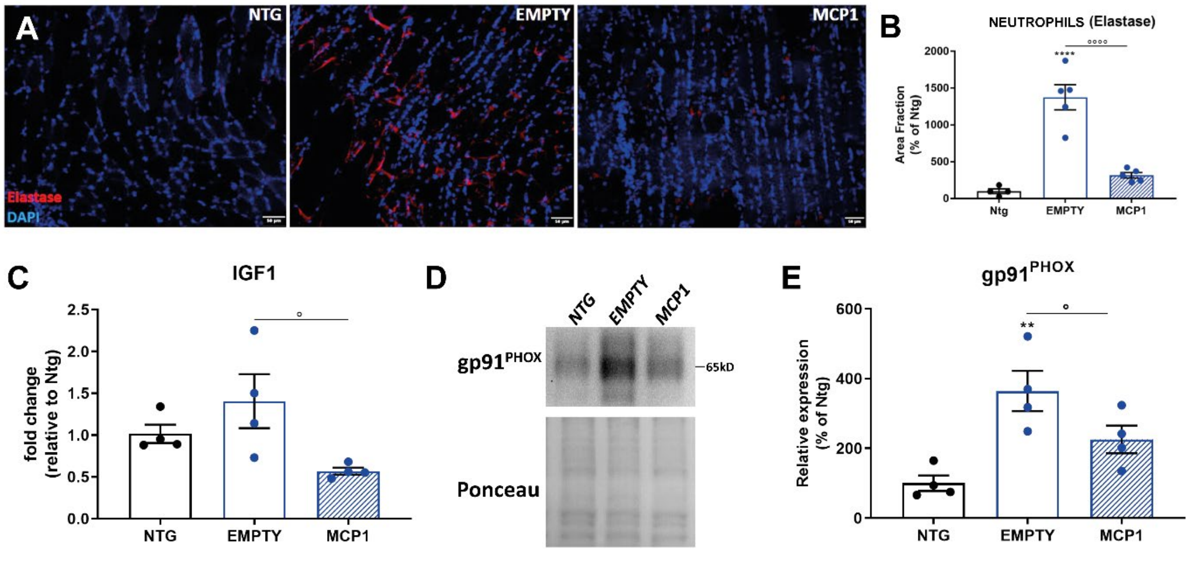

Figure S5_ ***The MCP1-mediated immune response dampens the inflammation in the skeletal muscle of C57SOD1^{G93A} mice.*** **A, B** Representative confocal micrographs and relative quantification of longitudinal sections of TA muscle of scAAV9_MCP1- and scAAV9(empty)-treated mice and Ntg littermates at 14 weeks stained with the neutrophils elastase enzyme (red) and DAPI (nucleus, blue). Scale bar, 50µm. Data are reported as mean±SEM of 3/5 serials sections per muscle from n=4 Ntg and n=5 SOD1^{G93A} mice per group. **C** Real-time PCR analysis of *Igf1* transcript in the TA muscle of scAAV9_MCP1- and scAAV9(empty)-treated mice compared to relative Ntg littermates at 20 weeks. Data are normalised to *β-actin* and expressed as mean±SEM. n=4 per experimental group. **D, E** Representative immunoblot images and relative densitometric analysis of gp91^{PHOX} expression in TA muscle extracts of scAAV9_MCP1- and scAAV9(empty)-treated mice and Ntg littermates at 20 weeks. Data are reported as mean±SEM. n=4 per experimental group. *p<0.05, **p<0.01, ***p<0.001, ****p<0.0001 Ntg Vs EMPTY or MCP1; °p<0.05, °°p<0.01, °°°°p<0.0001 EMPTY Vs MCP1 by one-way ANOVA with Fisher post-analysis.

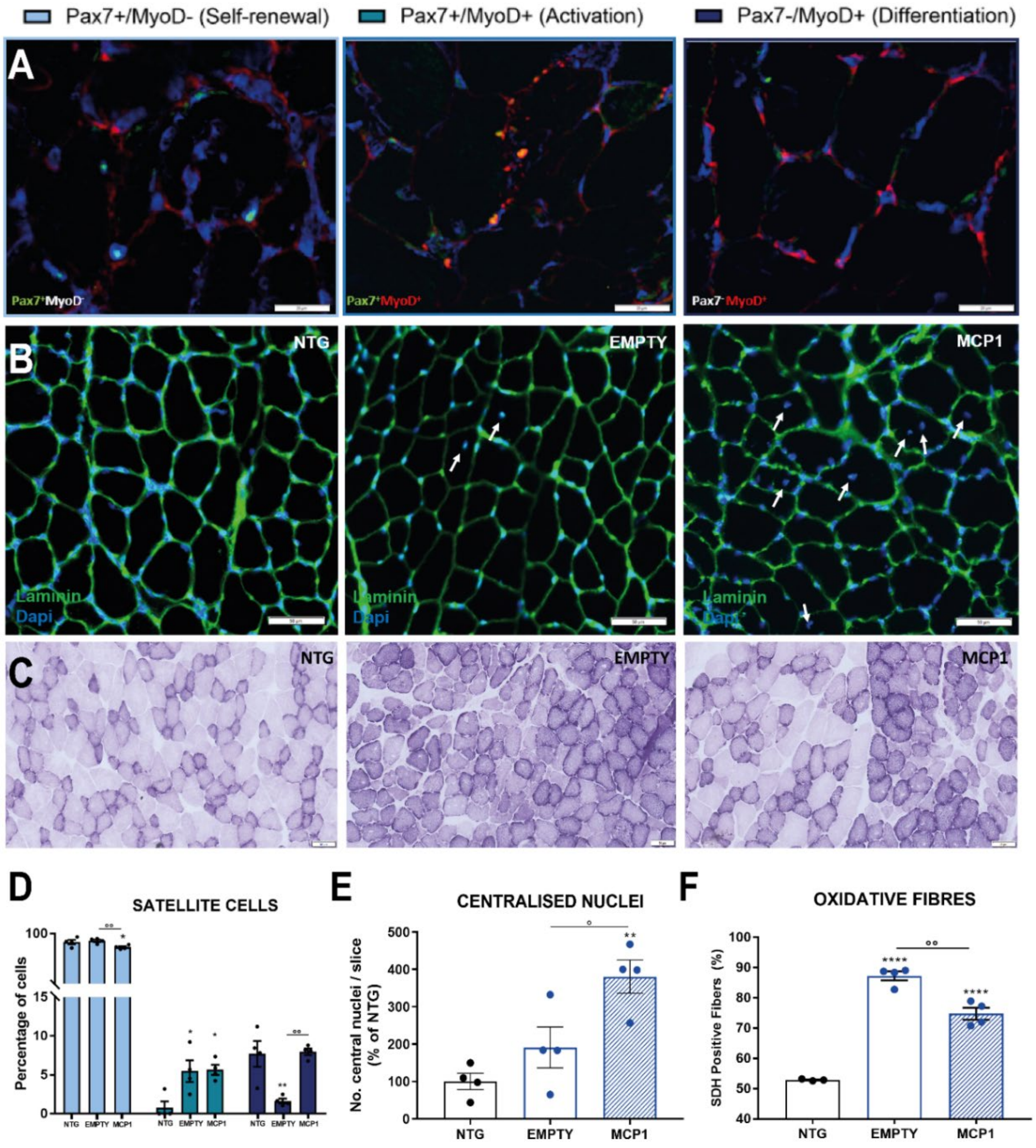

Figure S6_ ***The MCP1-mediated immune response triggers the myogenic programme, lessening myofibre metabolic dysregulation in C57SOD1^{G93A} mice.*** **A-C** Confocal micrographs of coronal sections of TA muscle stained with (A) Pax7 (green), MyoD (red) and DAPI (blue); (B) Laminin (green) and DAPI (blue) or (C) Succinate dehydrogenase enzyme of scAAV9_MCP1- and scAAV9(empty)-treated mice and Ntg littermates at 14 weeks. Scale bar, (A) 20µm; (B, C) 50µm. **D** Analysis of satellite cells dynamic in the TA muscle of scAAV9_MCP1-treated mice compared with the scAAV(empty) group. **E** Morphometric evaluation of centralised myonuclei in the TA muscle of scAAV9_MCP1-treated mice compared with the scAAV(empty) group. **F** Succinate dehydrogenase (SDH) histochemical analysis of the TA muscle of scAAV9_MCP1-treated mice compared with the scAAV9(empty) group. Data are reported as mean±SEM of 3/5 serial sections per muscle from n=4 mice per experimental group. *p<0.05, **p<0.01, ****p<0.0001 Ntg Vs EMPTY or MCP1; °p<0.05, °°p<0.01 EMPTY Vs MCP1 by or (D) two-way ANOVA or (E, F) one-way ANOVA with Fisher post-analysis.

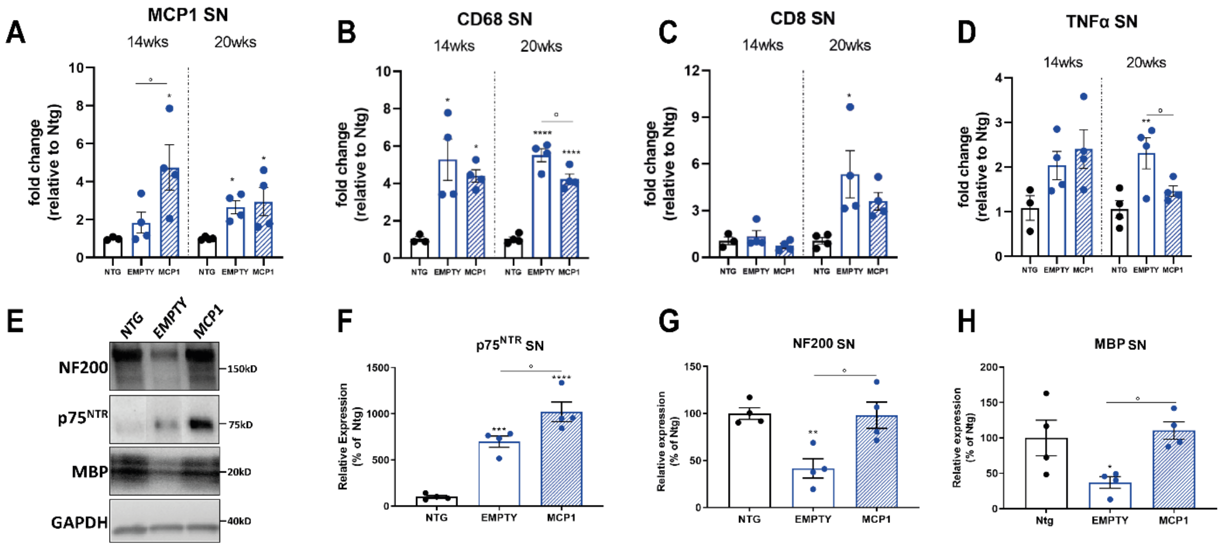

Figure S7_ ***The MCP1 boosting preserved motor axon from degeneration in the sciatic nerve of C57SOD1^{G93A} mice.*** **A-D** Real-time PCR analysis of (A) *Mcp1*, (B) *CD68*, (C) *CD8a* and (D) *TNFα* transcript in the sciatic nerve (SN) of scAAV9_MCP1- and scAAV9(empty)-treated mice compared to relative Ntg littermates. Data are normalised to *β-actin* and expressed as mean±SEM. 14 weeks: n=3 Ntg and n=4 SOD1^{G93A} mice per group; 20 weeks: n=4 per experimental group. **E-H** Representative immunoblot images and relative densitometric analysis of (E, F) p75^{NTR}, (E, G) NF200 and (E, H) MBP expression in SN extracts of scAAV9_MCP1- and scAAV9(empty)-treated mice and Ntg littermates at 20 weeks. Data are reported as mean±SEM. n=4 per experimental group. *p<0.05, **p<0.01, ***p<0.001, ****p<0.0001 Ntg Vs EMPTY or MCP1; °p<0.05, EMPTY Vs MCP1 by one-way ANOVA with Fisher post-analysis.

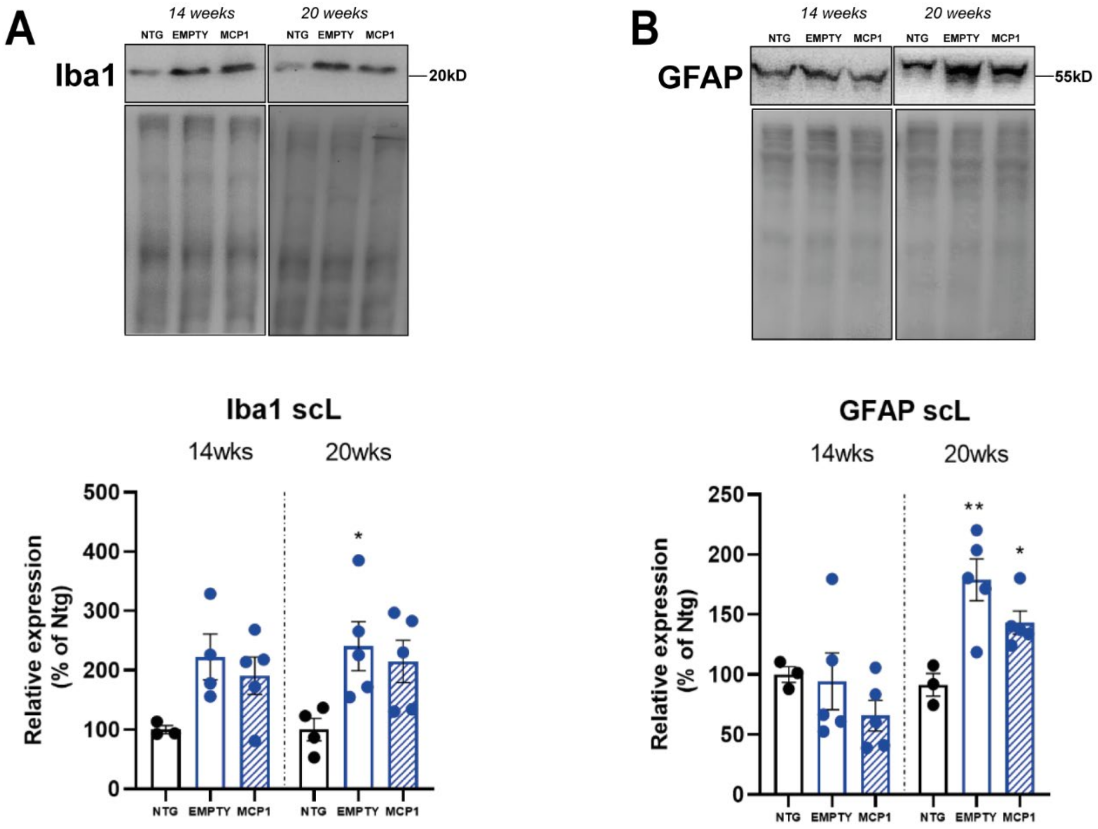

Figure S8_ ***The MCP1 boosting within MN perikarya does not affect astroglia proliferation in C57SOD1^{G93A} mice.*** Representative immunoblot images and relative densitometric analysis of (A) Iba1 (microglia) and (B) GFAP (astrocytes) expression in the lumbar spinal cord extracts of scAAV9_MCP1- and scAAV9(empty)-treated mice and Ntg littermates at 14 and 20 weeks. Data are reported as mean±SEM. n=3/5 per experimental group at each time point. *p<0.05, **p<0.01 Ntg Vs EMPTY or MCP1 by one-way ANOVA with Fisher post-analysis.

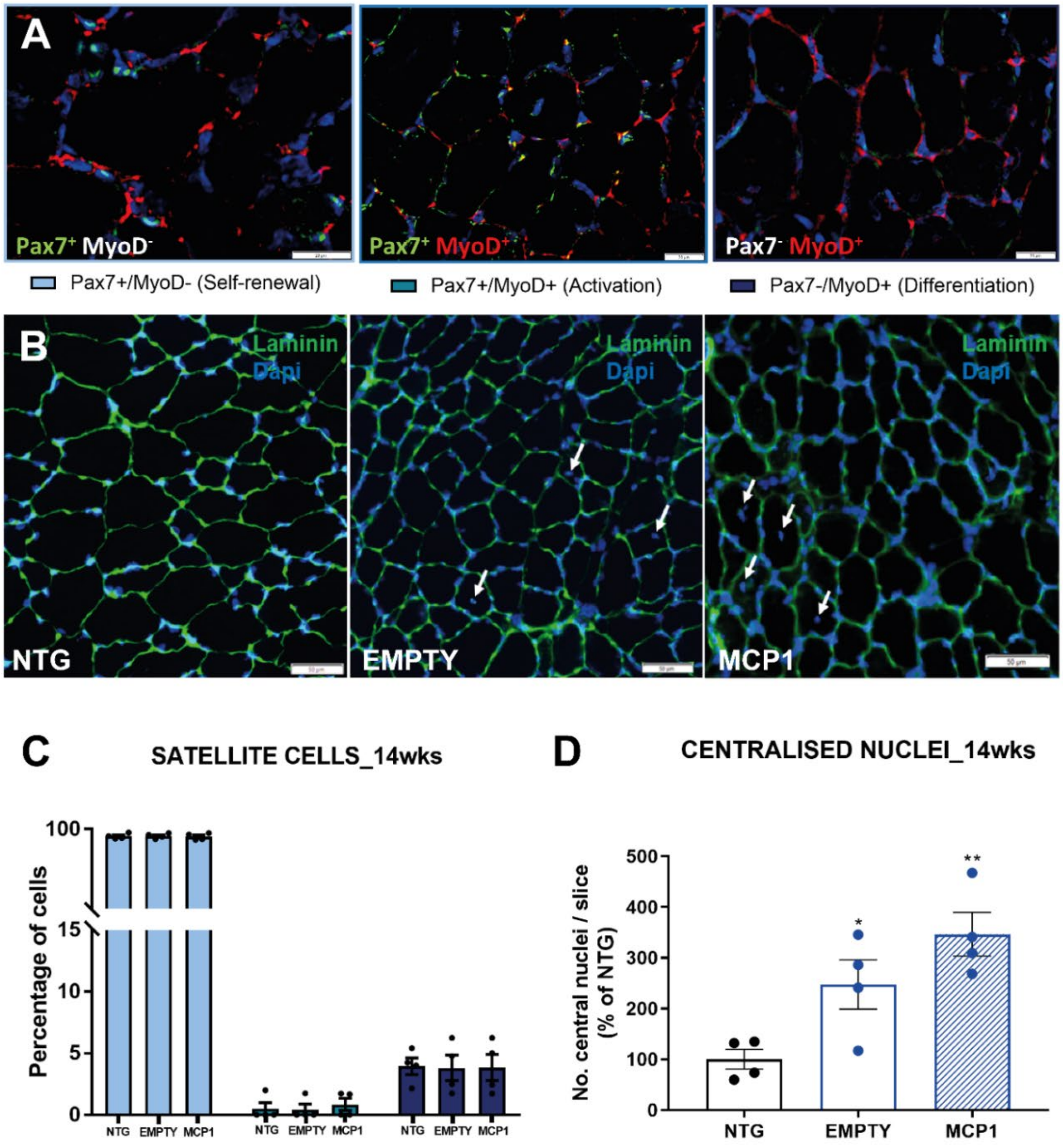

Figure S9_ *The scAAV9_MCP1 injection in C57SOD1^{G93A} mice does not promote an early forepaw muscle myogenesis.* **A, B** Confocal micrographs of coronal sections of TB muscle stained with (A) Pax7 (green), MyoD (red) and DAPI (blue) or (B) Laminin (green) and DAPI (blue) of pre-symptomatic scAAV9_MCP1- and scAAV9(empty)-treated mice and Ntg littermates (the same representative image of Figure 7E illustrates sel-renewal; activated and differentiating satellite cells). Scale bar, (A) 20µm; (B) 50µm. **C** Analysis of satellite cells dynamic in the TB muscle of scAAV9_MCP1-treated mice compared with the scAAV(empty) group at 14 weeks of age. **D** Morphometric evaluation of centralised myonuclei in the TB muscle of scAAV9_MCP1-treated mice compared with the scAAV(empty) group at 14 weeks of age. Data are reported as mean±SEM of 3/5 serial sections per muscle from n=4 mice per experimental group. *$p<0.05$, **$p<0.01$ Ntg Vs EMPTY or MCP1 by (C) two-way ANOVA or (D) one-way ANOVA with Fisher post-analysis.

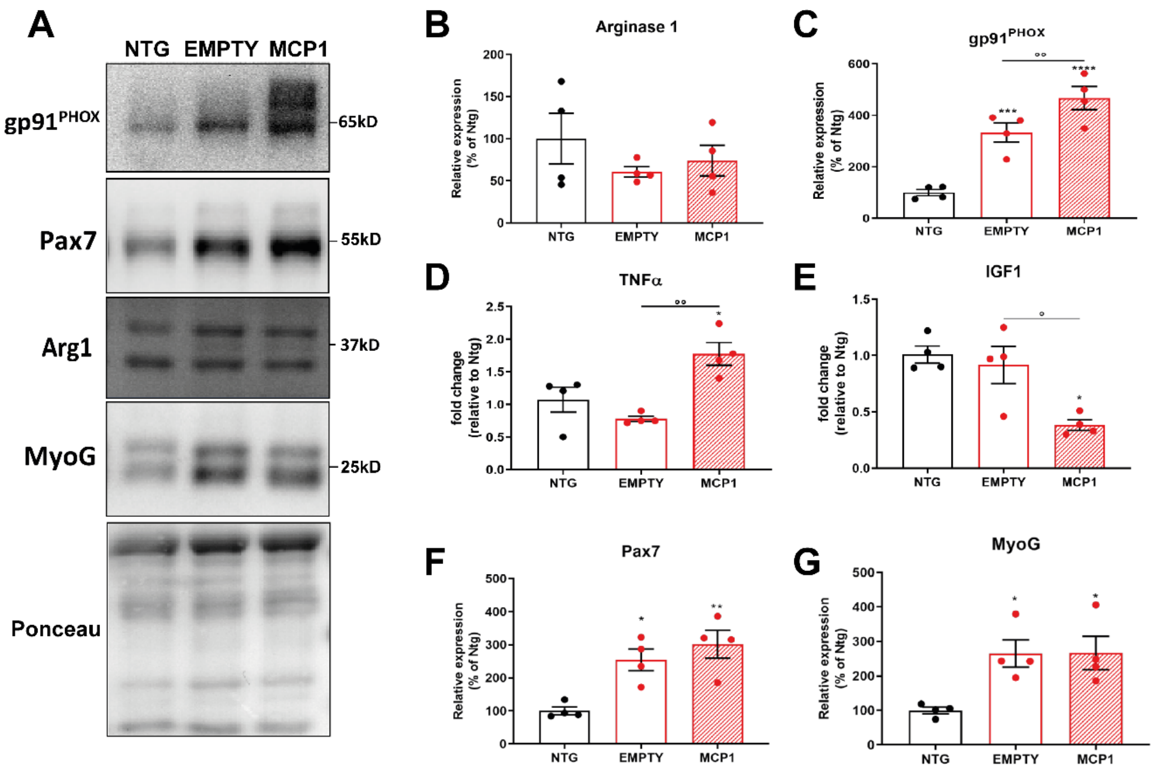

Figure S10_ ***The MCP1 boosting exacerbates the inflammation without triggering the myogenic programme in 129SvSOD1^{G93A} mice.*** **A** Representative immunoblot images of gp91^{PHOX}, Pax7, Arginase 1 and MyoG expression in TA muscle extracts of scAAV9_MCP1- and scAAV9(empty)-treated 129SvSOD1^{G93A} mice and Ntg littermates at 17 weeks. **B, C** The densitometric analysis does not show any difference between the two groups of 129SvSOD1^{G93A} mice in the (B) Arginase 1 expression; conversely, (C) gp91^{PHOX} results significantly upregulated in the hind paw muscle of scAAV9_MCP1-treated mice compared with the control group. **D, E** Real-time PCR analysis of (D) *Tnfα* and (E) *Igf1* transcript in the TA muscle of scAAV9_MCP1- and scAAV9(empty)-treated mice compared to relative Ntg littermates at 17 weeks. Data are normalised to β-actin and expressed as mean±SEM. **F, G** The densitometric analysis does not show any difference between the two groups of 129SvSOD1^{G93A} mice in the (F) Pax7 and (G) MyoG expression in the TA muscle. Data are reported as mean±SEM. n=4 per experimental group. *p<0.05, **p<0.01, ***p<0.001, ****p<0.0001 Ntg Vs EMPTY or MCP1; °p<0.05, °°p<0.01 EMPTY Vs MCP1 by one-way ANOVA with Fisher post-analysis.

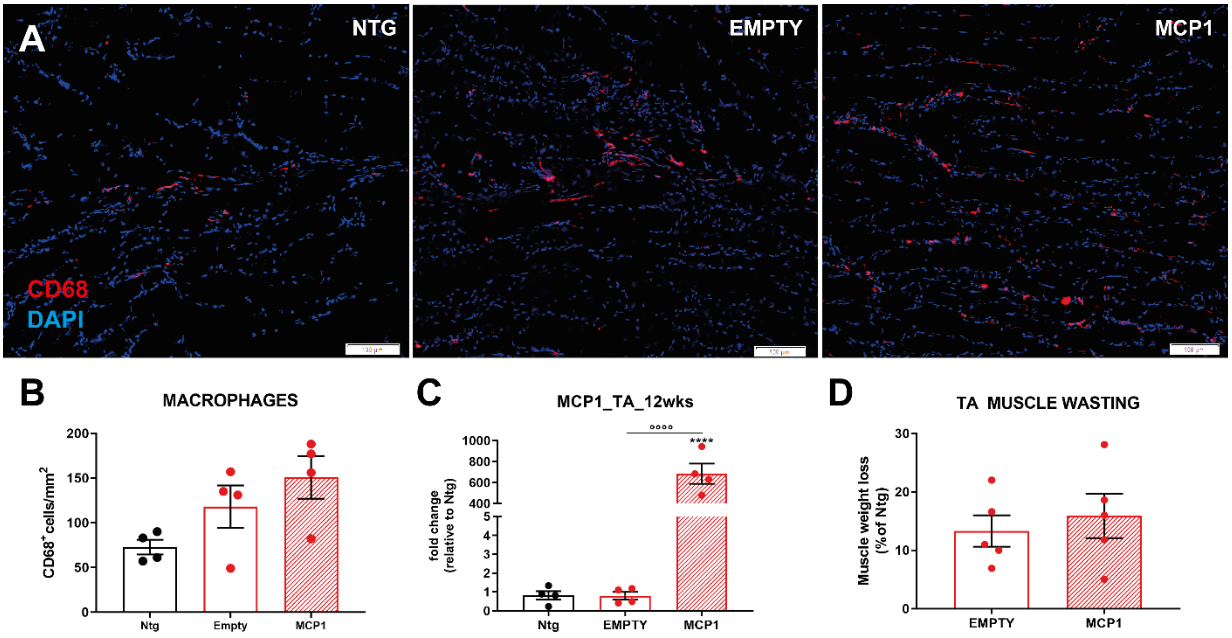

Figure S11_ ***129SvSOD1^{G93A} mice are insensitive to the muscular MCP1 boosting even at the pre-symptomatic stage.*** **A, B** (A) Representative confocal micrographs of longitudinal sections of TA muscle of scAAV9_MCP1- and scAAV9(empty)-treated mice and Ntg littermates at 12 weeks stained with the phagocytic marker CD68 (red) and DAPI (nucleus, blue). Scale bar, 100µm. (B) The relative quantification does not show any difference between scAAV9_MCP1- and scAAV9(empty)-treated 129SvSOD1^{G93A} mice in terms of macrophages recruitment in the TA muscle at the pre-symptomatic disease stage. Data are reported as mean±SEM of 3/5 serials sections per muscle from n=4 mice per experimental group. **C** Real-time PCR analysis of *Mcp1* transcript in the TA muscle of scAAV9_MCP1- and scAAV9(empty)-treated mice compared to relative Ntg littermates at 12 weeks. Data are normalised to β-actin and expressed as mean±SEM. n=4 per experimental group. **A** Muscle wasting was calculated by measuring the TA muscle weight of scAAV9_MCP1- and scAAV9(empty)-treated mice compared to relative Ntg littermates at 12 weeks of age. The percentage of muscle atrophy was calculated relative to Ntg mice. Data are reported as mean±SEM. n=5 per experimental group. ****p<0.0001 Ntg Vs MCP1; °°°°p<0.0001 EMPTY Vs MCP1 by one-way ANOVA with Fisher post-analysis.

Supplementary Table 1 Pro- and anti-inflammatory markers analysed

Marker	Polarisation
Arginase 1 (Arg1)	*anti-inflammatory*
Cytochrome b-245 heavy chain (gp91^{PHOX})	*pro-inflammatory*
inducible nitric oxide synthase (iNOS)	*pro-inflammatory*
Insulin-like Growth Factor 1 (Igf1)	*produced by M1-macrophages to trigger the M2-gene programme*
Interleukin 1β (Il1β)	*pro-inflammatory*
Interleukin 4 (Il4)	*anti-inflammatory*
Mannose receptor (CD206)	*anti-inflammatory*
Sirtuin 1 (Sirt1) deacetylase	*anti-inflammatory*
Tumour necrosis factor-α (Tnfα)	*pro-inflammatory*
